# MR-supported candidate genes and pathway analysis between ischemic stroke and osteoporosis: an integrated multi-omics study

**DOI:** 10.3389/fimmu.2026.1926168

**Published:** 2026-10-21

**Authors:** Xinyun Li, Yuchen Lou, Jingting Zhao, Zhenfei Xiong, Xiaoying Zhou

**Affiliations:** 1 Rehabilitation School, Hangzhou Medical College, Hangzhou, China; 2 Tongxiang City Gaoqiao Subdistrict Central Health Center, Tangxiang, China; 3 Hangzhou Medical College Affiliated Tongxiang Hospital Medical Group Gaoqiao Branch, Tangxiang, China; 4 Rehabilitation Department, Zhejiang Provincial People’s Hospital, Hangzhou, China; 5 Hand and foot surgery, Hangzhou Xiaoshan District Chinese Medicine Hospital, Hangzhou, China

**Keywords:** immune-metabolic, ischemic stroke, mechanisms, osteoporosis, shared molecules

## Abstract

**Background:**

The global prevalence of ischemic stroke (IS) and osteoporosis (OP) is rising, posing significant public health challenges in aging populations. We aimed to uncover shared genetic factors and elucidate the molecular mechanisms linking IS and OP to identify potential targets for integrated preventive and therapeutic interventions.

**Methods:**

A comprehensive multi-omics analysis was performed to assess associations and molecular pathways connecting IS and OP. Summary statistics for IS and OP were integrated with expression quantitative trait loci data from the eQTLGen consortium (n = 4,463) to identify common significant single nucleotide polymorphisms (SNPs) related to both diseases. Follow-up transcriptomic profiling and single-cell RNA sequencing were employed to investigate functional pathways, regulatory networks, and cell-type-specific expression patterns associated with these loci. Finally, the expression levels of shared molecules were confirmed using animal models, and subsequent functional analyses were conducted on these genes.

**Results:**

Two-sample Mendelian randomization identified *FCGR2B* and *CYBB* as exploratory MR-supported candidate genes with shared eQTL instruments. Transcriptome-wide analysis linked these loci to immune-metabolic pathways, including oxidative stress response (FDR = 0.003) and interferon-γ signaling (FDR = 0.01). Mouse-derived ischemic-stroke single-cell RNA-seq provided exploratory cell-type localization: *Fcgr2b* and *Cybb* were preferentially enriched in monocytes and macrophages (FDR < 0.05), whereas *Sirt1* was predominantly localized to endothelial cells and tissue stem cells in the diseased mouse microenvironment. Of note, the osteoporosis single-cell dataset (GSE147287) contained only one OP patient, and its cell-type patterns should therefore be interpreted as exploratory case observations rather than generalized findings. Regulatory network analysis identified the cisbp:M1399 transcription factor motif (NES = 4.12) with the highest enrichment score, which is predicted to separately regulate the transcription of *FCGR2B, CYBB* and *SIRT1*. Pathway enrichment analysis revealed metabolic reprogramming (AUC = 0.78, p=0.004) as a key mechanistic node differentiating high-and low-expression cell groups. The expression levels of the shared molecules were validated in a mouse model.

**Conclusions:**

This multi-omics study implicates immune-metabolic dysregulation as a shared pathological feature of ischemic stroke and osteoporosis. *FCGR2B*, *CYBB* and *SIRT1* are exploratory MR-supported candidate genes putatively associated with both diseases, offering potential clues for dual-purpose preventive and therapeutic strategies for age-related IS-OP comorbidity.

## Introduction

Osteoporosis (OP), a chronic skeletal disorder characterized by reduced bone mineral density (BMD) and elevated fracture risk, affects over 200 million individuals globally, with prevalence rising sharply in aging populations (1). Beyond its direct morbidity, OP is increasingly recognized for its systemic complications, including cerebrovascular disease. Notably, patients with OP exhibit a 1.8-fold increased risk of ischemic stroke (IS) (95% CI 1.4–2.3), while survivors of IS experience a ​7.3% decline in BMD within two years, accelerating fracture risk and perpetuating a cycle of disability (2). This bidirectional relationship is amplified by shared risk factors, including advanced age (comorbidity rate of 34% in adults >65 years), vitamin D deficiency (OR = 2.1), and chronic low-grade inflammation (CRP >3 mg/L) (2).

Ischemic stroke, the second-leading cause of death worldwide, imposes a parallel burden, with 12.2 million new cases annually (3). Despite advances in acute interventions, long-term outcomes remain poor due to recurrent events and complications exacerbated by OP-related frailty. For instance, post-stroke immobilization and OP-associated fractures synergistically elevate thrombosis risk, creating a feedback loop that worsens both conditions (4). However, the molecular mechanisms underlying this relationship remain unresolved. While observational studies highlight associations between OP, systemic inflammation, and IS risk, causal pathways bridging skeletal and cerebrovascular health are poorly defined (5).

Current evidence focuses on isolated risk factors such as vitamin D deficiency or physical inactivity, neglecting integrative analyses of shared genetic and cellular drivers. Addressing this gap is critical for developing therapies that disrupt the OP-IS cycle. In this study, we employed Mendelian randomization to infer causality, transcriptomic networks to map shared pathways, and single-cell profiling to resolve cellular drivers of OP-IS comorbidity. Our objectives are to prioritize exploratory candidate genes putatively associated with both OP and IS risk and define overlapping immune-metabolic pathways mediating their bidirectional correlation, to nominate potential dual-purpose intervention targets for high-risk populations.

## Methods

### Data sources

#### Genetic and transcriptomic data acquisition

Genetic association summary statistics for osteoporosis (OP) and ischemic stroke (IS) were obtained from FinnGen (finngen_R10_M13_OSTEOPOROSIS; 8 017 cases, 391 037 controls) and the EBI GWAS Catalog (ebi-a-GCST90018864; 11 929 IS cases, 472 192 controls), respectively. To screen for MR-supported putative candidate genes, we incorporated blood-derived cis-eQTL data from the eQTLGen Consortium Phase II (31 684 samples), together with a set of 4 463 druggable genes (6). This druggable gene set was compiled from two public drug-target resources: DGIdb (version 4.0) and Open Targets Platform (release 22.02). The druggable-gene list served as an exploratory screening dimension to prioritize genes with potential translational therapeutic value. We acknowledge that filtering against this druggable gene set may introduce selection bias; importantly, druggable annotation was combined with MR causal estimates, eQTL evidence and single-cell expression signatures and was not the sole inclusion criterion for hub-gene selection. Notably, the GWAS summary statistics and blood cis-eQTLs used for candidate screening originate entirely from human peripheral-blood samples. By contrast, single-cell RNA-seq datasets profiled cell-type-specific expression in brain (ischemic stroke) and bone marrow (osteoporosis), whereas *in-vivo* mouse validation measured transcript abundance in peripheral blood. Given strong tissue-specificity of gene expression and cis-regulatory effects, eQTL variants detected in circulating blood immune cells cannot fully represent regulatory activity in tissue-resident cell populations such as brain endothelial cells, microglia, osteoblasts and osteoclasts. Although we leveraged brain and bone-marrow single-cell data to map candidate-gene expression within target lesion tissues and partially mitigate this limitation, blood-based eQTL signals cannot be directly extrapolated to these tissue-resident cell subsets. All analytical scripts for blood-based GWAS/eQTL, bulk transcriptome and tissue single-cell RNA-seq analyses are deposited in **Supplementary Codes** to enable full reproducibility of our tissue-aware analytical workflows (6, 7).

For transcriptomic profiling, bulk RNA-seq data from the Gene Expression Omnibus (GEO) included GSE58294 (92 participants: 23 controls, 69 IS), GSE56815 (80 participants), in which samples were stratified into high-BMD and low-BMD subgroups for osteoporosis-related transcriptome analysis, annotated under GPL570 and GPL96 platforms. Single-cell RNA-seq data (GSE174574 for IS and GSE147287 for OP) were processed to characterize immune and cellular heterogeneity. The raw data processing included quality control, removal of low-expressing genes (CPM> 1), and normalization using the limma-voom workflow (4, 5).

#### Data quality control

All GWAS summary statistics underwent standard quality control (QC) procedures, including the exclusion of single nucleotide polymorphisms (SNPs) with genotype dropout rates> 0.05 and minor allele frequencies (MAF) <0.01, as well as variants failing the Hardy-Weinberg equilibrium test (*P* < 1×10^-8^) (8).

Gene mapping strategy: To identify candidate genes from GWAS summary data, we employed a position-based mapping strategy (genomic coordinates ± 10 kb). Subsequently, we integrated blood cis-eQTL data from the eQTLGen Consortium (Phase II, 31,684 samples) to provide functional evidence. The initial MR exploratory analysis focused on 4,463 druggable genes, which were assembled from DGIdb (version 4.0) and Open Targets Platform (release 22.02) (1, 2).

#### Causal inference framework​​

To evaluate whether OP influences IS risk through shared molecular pathways, we performed two-sample Mendelian randomization (MR). Instrumental variables (IVs) were selected as eQTLs (*P* < 1×10^-8^) for druggable genes, with linkage disequilibrium (LD) clumping (R² < 0.001; window = 10,000 kb) to exclude correlated variants. Four MR methods—Inverse-variance weighted (IVW), MR-Egger, Weighted Median, and Weighted Mode—were applied to estimate causal effects. Sensitivity analyses, including Cochran’s Q test for heterogeneity and MR-Egger intercepts for pleiotropy, were conducted. Outlier SNPs were iteratively excluded, and recalculated effect sizes assessed robustness.

For GWAS summary-data preprocessing, SNPs were filtered based on minor-allele frequency > 0.01, imputation quality score INFO > 0.8. LD clumping was performed with r² = 0.001 and clumping distance window 10000 kb. All MR analyses were performed using TwoSampleMR (R package, version 0.5.6). A Bonferroni-corrected *P*-value threshold was adopted to define significant MR signals.

All results of multiple regression analysis, including effect estimates (OR), standard errors, 95% confidence intervals, *P*-values, and heterogeneity and pleiotropy test indicators, are fully provided in the **Supplementary Tables**.

The threshold(*P* < 5×10^-5^) was applied for filtering cis-eQTL SNPs to construct MR instrumental variables, rather than serving as the Bonferroni-corrected significance threshold for gene-level MR causal testing across the 4463 druggable genes. This SNP-filtering threshold was adopted according to a previously published MR study (3). For large-scale exploratory MR screening of druggable genes, we used a nominal IVW *P* < 0.05 as exploratory screening threshold to prioritize candidate genes for downstream validation (9).

#### Colocalization analysis

To evaluate whether key-gene loci harbor shared causal variants underlying ischemic stroke and osteoporosis, genetic variants within 1 Mb upstream and downstream of each gene probe were extracted and matched against GWAS summary statistics of ischemic stroke (GCST90018864) and osteoporosis (FinnGen R10, M13_OSTEOPOROSIS). After genomic coordinate and allele harmonization, Bayesian colocalization analysis was performed using the coloc.abf function from the R package *coloc*, with parameters set as *p*1 = 1×10^-4^, *p*2 = 1×10^-4^, and *p*12 = 1×10^-5^. Under the assumption that at most one causal variant exists per genomic region for each trait, regional posterior probabilities and SNP-level SNP.PP.H4 values were calculated to identify top candidate variants with the highest conditional posterior probability. SNP.PP.H4 denotes the posterior probability that a given SNP represents the shared causal variant under the hypothesis of shared causality (10).

### Functional and pathway analysis​​ ​​

#### Gene set enrichment​​

Gene Set Variation Analysis (GSVA) was implemented to evaluate pathway activity shifts between IS and OP cohorts using the Molecular Signatures Database (MSigDB v7.0). Gene sets were ranked by differential expression, and non-parametric kernel-based permutation tests (1,000 iterations) identified pathways enriched in disease states (FDR < 0.05). Gene Set Enrichment Analysis (GSEA) further stratified patients by hub gene expression (high vs. low), comparing pathway activity through pre-ranked gene lists (FDR < 0.05). We clarify that ‘enrichment’ refers to the overrepresentation of a set of genes in a specific pathway, not a single gene (5). GSEA classifies patients into high-expression and low-expression groups based on the median expression levels of hub genes. This grouping is justified by the assumption of a bimodal distribution in gene expression, which allows for the exploration of potential threshold effects in their biological functions, and facilitates comparison of pathway activity between the two groups (11–13).

#### Immune microenvironment profiling​​

Immune cell infiltration was quantified using single-sample gene set enrichment analysis (ssGSEA), leveraging 547 immune-specific markers to estimate abundances of T cells, B cells, myeloid subsets, and plasma cells. CIBERSORTx refined deconvolution via support vector regression, correlating immune cell proportions with hub gene expression (Pearson’s r).

### Transcriptional regulation and single-cell analysis​​ ​​

#### Motif enrichment and transcription factors​​

RcisTarget identified transcription factors (TFs) regulating hub genes by calculating normalized enrichment scores (NES) for motif pairs. Motifs were annotated through sequence similarity and database integration, with AUC-based prioritization (NES > 3.0) indicating significant regulatory activity.

#### Single-cell RNA-seq processing​​

To bridge the gap between human genetic discoveries and downstream *in-vivo* murine validations, two distinct single-cell datasets were used with separate processing pipelines. GSE174574 is a mouse-derived brain single-cell dataset for ischemic-stroke research. Mouse gene identifiers from GSE174574 were converted to human homologous gene symbols using the biomaRt package (14, 15), and interspecies extrapolation limitations are acknowledged throughout interpretation. By contrast, GSE147287 is human-origin bone-marrow-derived single-cell data for osteoporosis; no homolog conversion was needed for this human dataset. Notably, the GSE147287 dataset comprises only one osteoporosis patient together with one osteoarthritis patient. Given the lack of multiple independent OP patient samples, pooled cell populations from this single case were applied exclusively for preliminary exploratory cell-type expression analysis, and this dataset does not possess sufficient sample size to capture population-wide transcriptomic heterogeneity among osteoporosis patients. Given the lack of multiple independent OP patient samples, pooled cell populations from this single case were applied exclusively for preliminary exploratory cell-type expression analysis, and this dataset does not possess sufficient sample size to capture population-wide transcriptomic heterogeneity among osteoporosis patients. The primary objective of our single-cell analysis was to characterize cell-type-specific expression patterns of candidate hub genes rather than conducting sample-level statistical inference between disease and control groups; all downstream analyses were performed at the cellular level, and individual cells were not treated as independent statistical observations to avoid pseudoreplication. Because these datasets were mainly deployed for cell-type localization of target genes, sample-level effects from individual mice or patients were not incorporated into the analytical model. Differential expression analysis was carried out using the limma statistical model with Bonferroni correction for multiple-testing adjustment, and results generated from single-cell data should be interpreted as cellular expression signatures instead of conclusive sample-based intergroup comparisons. For preprocessing, Seurat (v4.3.0) was used to filter low-quality cells (< 3 counts per gene) and normalize data via SCTransform. Following dimensionality reduction by PCA with 30 components, unsupervised clustering (resolution = 0.6) and t-SNE visualization were performed, and clusters enriched in disease cases were annotated for immune or metabolic cell lineages.

#### Animals and confirmation

All animal care procedures during the experiment were in accordance with the guidelines established by the Experimental Committee of the People’s Hospital of Hangzhou Medical College, and have been approved by the Animal Room Ethics Committee of Hangzhou medical college (Approval No: 202502049). Ten female mice, each aged 8 weeks, were acquired from the Animal Laboratory Center of Hangzhou Medical College and were housed at the Hangzhou Medical College. The mice were divided into groups of five per cage, supplied with water and food, and subjected to a 12-hour light/dark cycle.

### Construction of mouse models for ischemic stroke and osteoporosis

Female C57BL/6J mice were first maintained under standard housing conditions for 8 months to establish aging-related baseline bone loss. Subsequently, transient middle cerebral artery occlusion (MCAO) surgery was performed to induce ischemic stroke. After MCAO operation, mice were kept for another 8-week observational period. During this post-stroke phase, reduced physical activity and limb disuse secondary to cerebral ischemic injury further aggravated bone deterioration. Finally, Micro-CT and TTC staining were conducted for bone microstructure assessment and cerebral infarction verification, respectively (**Supplementary Materials 1**–**3**).

Of note, this model did not adopt ovariectomy or other dedicated osteoporosis-inducing surgical manipulations. The osteoporosis-like phenotype arises from the combined effects of pre-existing age-dependent bone loss and post-stroke disuse-mediated bone deterioration triggered by MCAO-induced brain injury. This experimental setting was selected to mimic clinical real-world scenarios in which elderly patients suffer new-onset ischemic stroke and subsequently develop accelerated bone loss.

In the current animal validation system, peripheral blood qPCR is adopted as the major transcriptional detection index. TTC staining and Micro-CT measurements are only performed to confirm successful establishment of MCAO and osteoporosis models, and all relevant imaging and quantitative data are deposited in **Supplementary Materials**.

### Real-time fluorescence quantitative PCR

Quantitative real-time PCR was performed using archived RNA aliquots from the existing mouse cohort (Control n=4, IS/OP n=4). The primer sequences of RNA are presented in **Table 1**. Independent technical replication was conducted by repeating qPCR assays on these stored RNA samples. Relative gene expression was calculated by the 2^-ΔΔCt method: ΔCt = Cq(target gene) − Cq(GAPDH). The mean ΔCt across all control samples was used as the calibrator, so that the average relative expression of the control group was normalized to 1. Individual biological replicates naturally fluctuate around this value. Raw Cq values are listed in **Supplementary Table S1**; amplification and melting curves are shown in **Supplementary Figure S10**.

**Table 1:** Primer sequences for co-susceptible genes (custom-designed).

Primer	Sequence(5’ to 3’)
Fcgr2b-F	TCCCAGATCCCTGTGAACAA
Fcgr2b-R	TTGACCACCAGGATGTCCTC
Cybb-F	GGCTGGAGTGGAGAATGTGA
Cybb-R	CGATGGCCTTCATTGTACCT
Sirt1-F	CAGTCCTTCCAGCACATAGG
Sirt1-R	ACATTGAGCACCAGCAAACA

### The overexpression and knockdown of Sirt1 in mice models

To functionally validate the role of Sirt1 in ischemic stroke and osteoporosis mice, we performed Sirt1 knockdown and overexpression experiments. Following the establishment of the post-stroke osteoporosis model, mice were randomly divided into three groups (n = 5 per group): PBS-injected(PBS), Sirt1 knockdown (KD) group, Sirt1 overexpression (OE) group. *SIRT1* knockdown and overexpression were achieved via intracerebroventricular injection of specific adeno-associated virus (AAV) vectors targeting Sirt1 or its negative control. Successful manipulation was confirmed by Western blot analysis measuring Sirt1 protein levels in the prefrontal cortex (**Supplementary Material 4**). Serum was collected to detect bone metabolic markers in mice. The serum was extracted from mice and the concentrations of bone-derived alkaline phosphatase(ALP) and C-terminal telopeptide of type I collagen(CTX-I) in the serum were detected by enzyme-linked immunosorbent assay (ELISA) using specific antibodies.

Notably, Mendelian-randomization candidate screening relied exclusively on blood-derived cis-eQTL data, which carries inherent tissue-specificity constraints that may limit direct extrapolation to brain- and bone-resident cell populations.

### Statistical analysis​​

The three core assumptions of MR analysis (relevance, exclusion restriction, monotonicity) were strictly tested. MR assumptions—relevance (IV-gene association), exclusion restriction (IV-outcome independence), and monotonicity—were rigorously tested. All computations were performed using R (v4.2.2) together with Bioconductor packages. For MR instrumental variant selection, we applied a SNP-level filter of (*P_eQTL_* < 5x10^-5^) following Woolf et al. (3). For exploratory gene-level MR screening across druggable genes, nominal IVW *P* < 0.05 was used for initial candidate prioritization. No gene-level Bonferroni correction (0.05/4463 ≈1.1×10-5) was enforced for the initial screening step, as this analysis aimed to generate candidate genes for subsequent multi-omics and experimental validation rather than drawing definitive causal conclusions solely from MR screening results.FDR < 0.05 was applied for pathway enrichment analyses, and two-tailed *P* < 0.05 was considered statistically significant for correlation analyses.

We thank the reviewer for the valuable suggestion. Following this recommendation, we performed FDR (Benjamini-Hochberg) correction on the IVW *P*-values of all 4,463 tested genes. As expected given the scale of multiple testing (4,463 genes), no gene remained significant after strict FDR correction, indicating that individual cis-eQTL-driven genetic effects on these two traits are of small magnitude and are readily masked by genome-wide multiplicity adjustment.

We would like to clarify that, in our study, MR served as a discovery step rather than a definitive causal-effect estimator. Given the polygenic, small-effect nature of the genetic instruments involved, applying a stringent genome-wide FDR threshold at this screening stage is methodologically over-conservative and would discard biologically plausible candidates before downstream validation. We therefore retained genes with nominal IVW *P* < 0.05 as the MR screening set, and relied on the subsequent multi-omics integration (bulk transcriptomics, single-cell analysis, transcription-factor network) and *in vivo* functional validation to control false positives and independently confirm the candidates. This MR screening + multi-layer functional confirmation paradigm has been widely adopted in recent MR-based multi-omics studies (16).

All statistical outputs are reported using adjusted *P* values. Nominal raw *P* values are not presented as primary results in the revised manuscript.

## Results

This study followed the workflow outlined in **Figure 1**. Genetic evidence in the present work was derived from three complementary levels: GWAS summary statistics captured disease-related genetic variants; eQTL datasets informed regulatory effects of SNPs on gene expression; and eQTL-based MR was applied to evaluate whether genetically predicted gene expression exerted potential causal influence on disease risk. *FCGR2B, CYBB*, and *SIRT1* were defined as shared MR-positive candidate genes, since significant MR signals were observed for both ischemic stroke and osteoporosis. Notably, the corresponding SNPs (rs1801274 for *FCGR2B*, rs6610656 for *CYBB*, rs3818292 for *SIRT1*) function as eQTL instrumental variables for MR, rather than shared disease-associated loci directly overlapped from GWAS.

**Figure 1:**
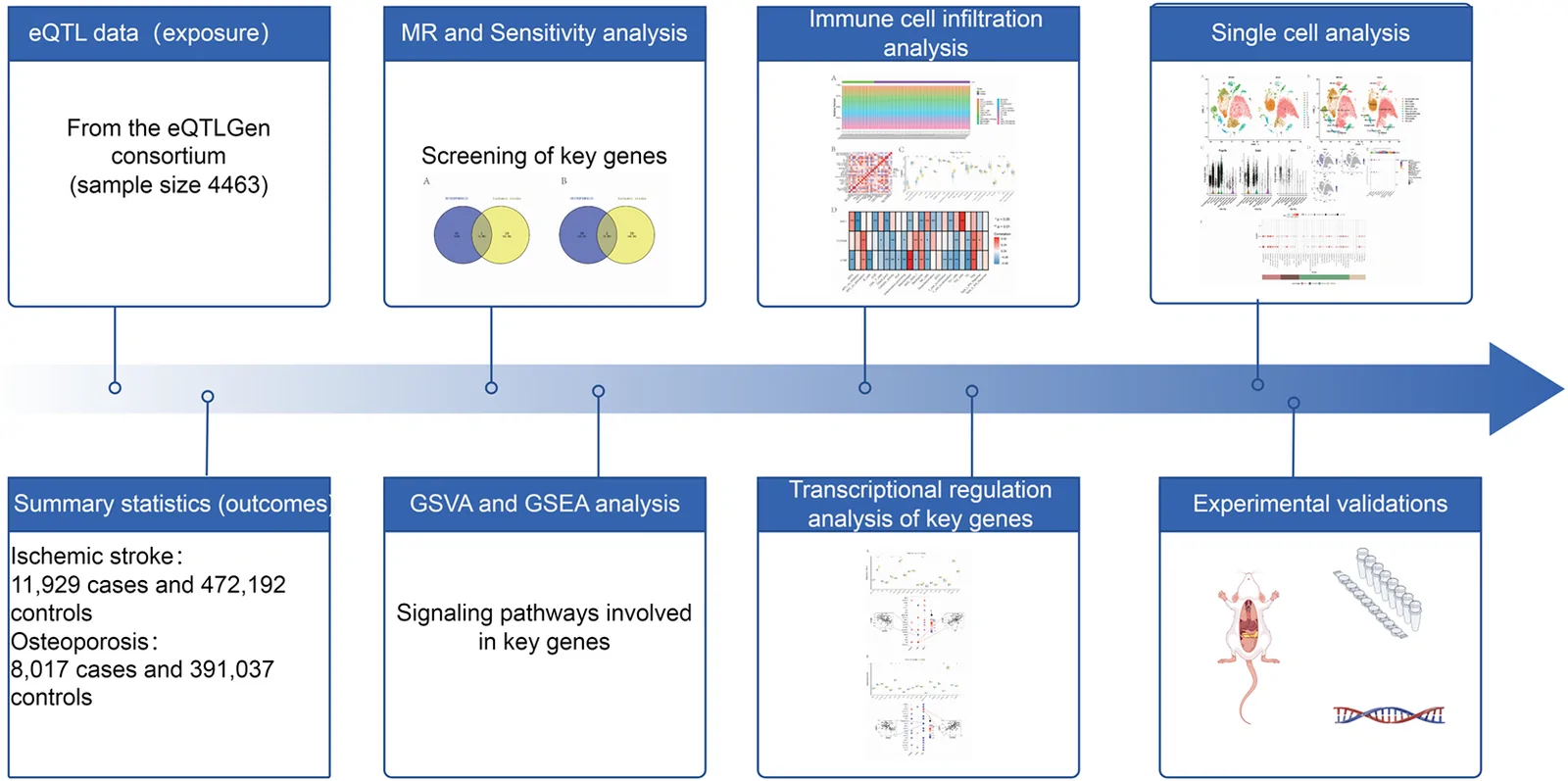
Workflow of the present study. Schematic overview illustrating the overall analytical pipeline for identifying shared susceptibility genes for ischemic stroke (IS) and osteoporosis (OP). Human multi-omics data including GWAS, eQTL and single-cell transcriptome datasets were integrated to screen candidate shared genes. Subsequent bioinformatic enrichment analyses were performed, followed by in-vivo qPCR validation using mouse peripheral-blood samples.

### Identification of candidate genes

To identify genes associated with ischemic stroke, we obtained outcome data from a GWAS meta-analysis of 484,121 individuals (11,929 cases and 472,192 controls; outcome ID: ebi-a-GCST90018864). Genetic variants (SNPs) significantly associated with each gene (*P* < 1e-8) were extracted using the extract_instruments and extract_outcome_data functions. After clumping SNPs to reduce linkage disequilibrium (R²< 0.001, window = 10,000 kb) and applying a secondary eQTL-SNP p-value threshold (P_2_<5x10^-5^) for instrument selection, 77 gene-exposure pairs were retained for MR analysis. We then applied a nominal IVW *P* < 0.05 as exploratory screening threshold for MR outcome testing (**Figure 2A**), which revealed several genes potentially associated with ischemic stroke risk. Genes such as KCNJ2 (OR = 0.913, 95% CI: 0.857–0.974, *P* = 0.006) and AKR1B1 (OR = 0.935, 95% CI: 0.890–0.983, *P* = 0.008) were linked to a reduced risk, while others such as DHRS13 (OR = 1.094, 95% CI: 1.034–1.157, *P* = 0.002) and SIK1 (OR = 1.156, 95% CI: 1.052–1.270, *P* = 0.003) were associated with increased risk (**Figure 2A**).

**Figure 2:**
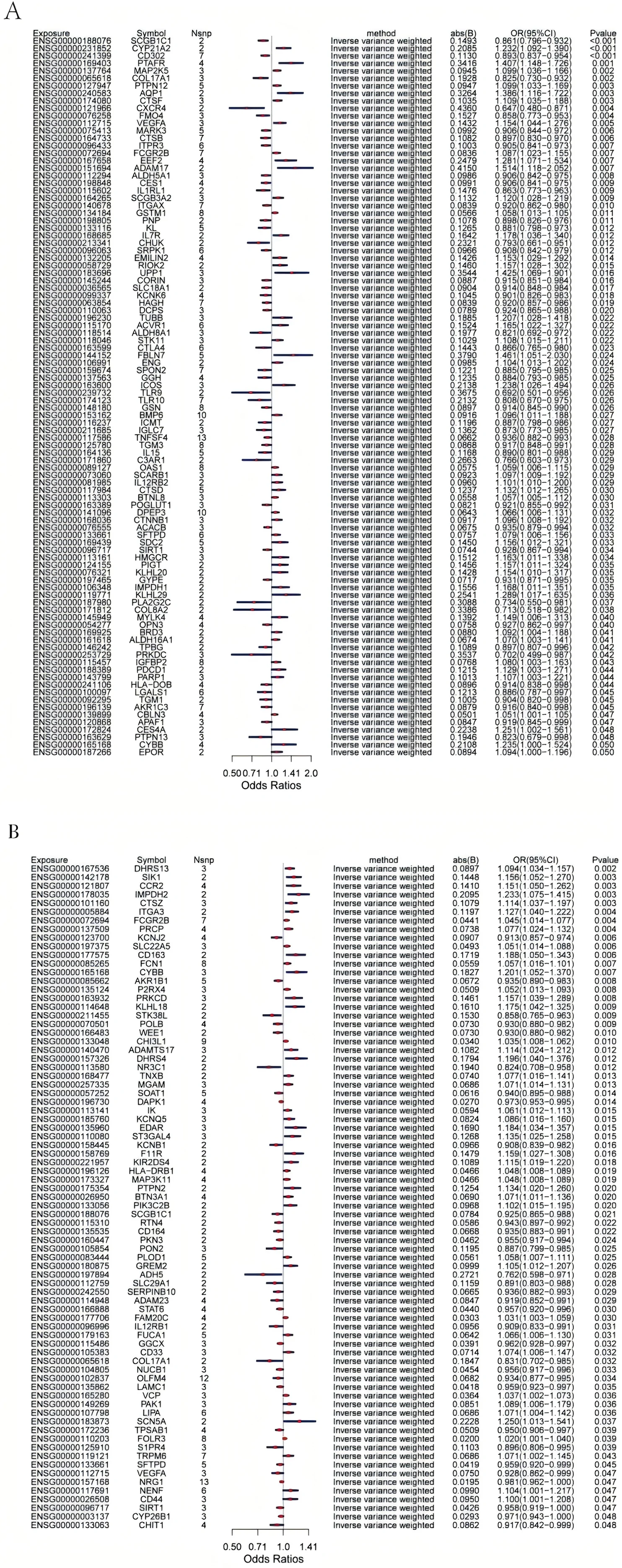
Genetic correlation and overlap analysis between ischemic stroke and osteoporosis. **(A)** the forest plot of ischemic stroke. **(B)** the forest plot of osteoporosis.

Heterogeneity tests showed no evidence of heterogeneity across the 77 causal associations (Q_pval > 0.05), and leave-one-out sensitivity analysis confirmed the robustness of 51 gene associations. These 51 genes were retained as candidate genes for ischemic stroke.

For osteoporosis, summary statistics were obtained from 399,054 individuals (8,017 cases and 391,037 controls; outcome ID: finngen_R10_M13_OSTEOPOROSIS). The same pipeline for SNP extraction, clumping, and instrument selection was applied, yielding 96 gene-exposure pairs subjected to MR analysis.

Genes such as SCGB1C1 (OR = 0.861, 95% CI: 0.796–0.932, *P* < 0.001) and CD302 (OR = 0.893, 95% CI: 0.837–0.954, *P* < 0.001) were associated with decreased osteoporosis risk, while CYP21A2 (OR = 1.232, 95% CI: 1.092–1.390, *P* < 0.001) and PTAFR (OR = 1.407, 95% CI: 1.148–1.726, *P* = 0.001) showed increased risk (**Figure 2B**). After excluding LGALS1 and FBLN7 due to heterogeneity (Q_pval < 0.05), 94 associations remained. Sensitivity analysis confirmed the robustness of 64 of these, which were retained as candidate genes for osteoporosis.

To identify genes involved in both ischemic stroke and osteoporosis, the causal gene sets from both conditions were intersected. Three independent genes—*FCGR2B* and *CYBB* (shared risk-associated MR signals),and *SIRT1* (a shared protective MR signal) were prioritized as exploratory candidates showing nominal association with both diseases (**Figure 3**). We regarded these three candidate genes as core molecular markers for subsequent multi-omics functional exploration. Comprehensive MR causal estimates for these three core genes for both ischemic stroke and osteoporosis outcomes, including odds ratios (OR), 95% confidence intervals, adjusted P-values, Cochran’s Q-statistic for heterogeneity, and MR-Egger intercept-based horizontal pleiotropy diagnostics are summarized in **Supplementary Tables S2**, **3**. Of note, these gene-level MR associations were derived from nominal uncorrected IVW *P*-values across 4463 druggable genes, and none remained significant after Benjamini-Hochberg FDR correction.

**Figure 3:**
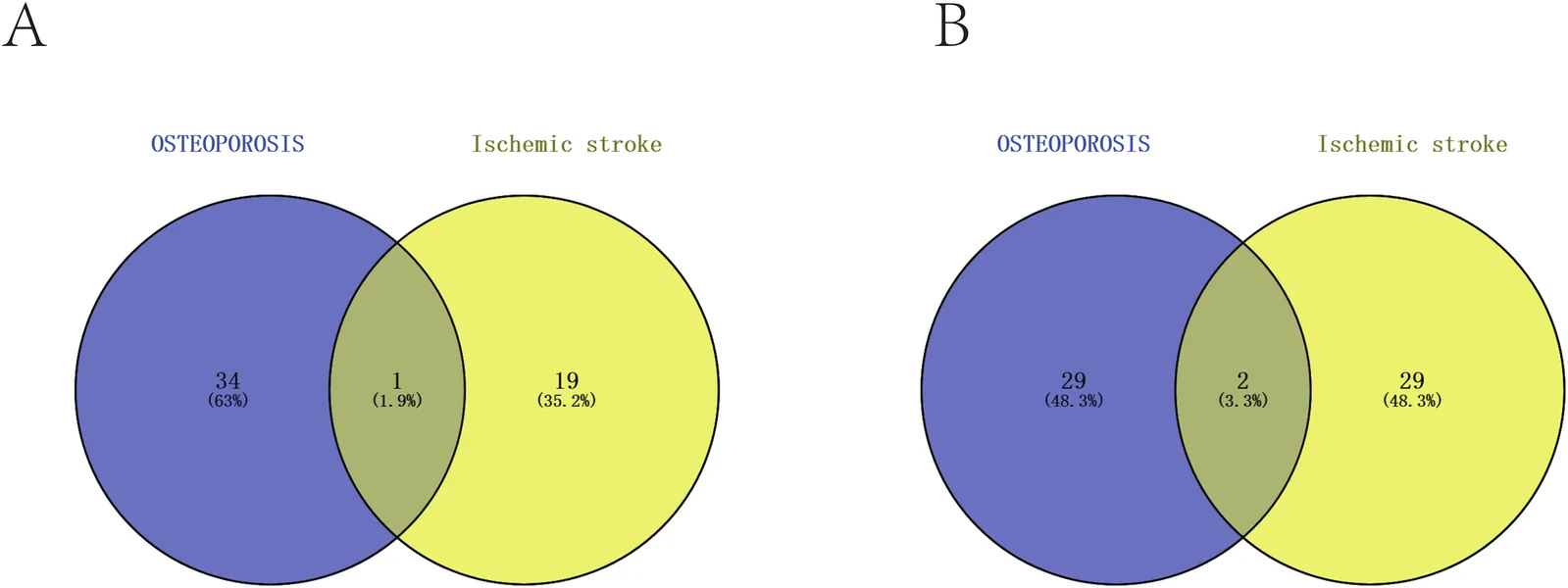
The key genes for downstream analyses. **(A)** Preliminary MR-derived candidate genes: 34 osteoporosis-specific, 19 ischemic-stroke-specific, 1 shared gene. **(B)** Robust candidates after sensitivity filtering: 29 osteoporosis-specific, 29 ischemic-stroke-specific, 2 shared genes. Among shared genes, *FCGR2B* and *CYBB* are shared risk loci, and *SIRT1* is the shared protective locus selected for follow-up validation.

### Colocalization analysis

Colocalization analyses were performed for *FCGR2B* and *SIRT1* with both ischemic stroke and osteoporosis. For *FCGR2B*, the top variant with the highest SNP.PP.H4 was rs61803028 for both diseases, with near-unity conditional posterior probabilities. For *SIRT1*, the leading variant was rs10997832; its SNP.PP.H4 values were 0.5503 for ischemic stroke and 0.5489 for osteoporosis, respectively. These findings indicated that under the hypothesis of shared causal variants, posterior probability was strongly concentrated at rs61803028 for *FCGR2B*, whereas probability distribution was relatively dispersed across the *SIRT1* locus (**Figure 4**). No high SNP.PP.H4 signals were observed for *CYBB*, and its full colocalization outputs are provided in **Supplementary Table 4**.

**Figure 4:**
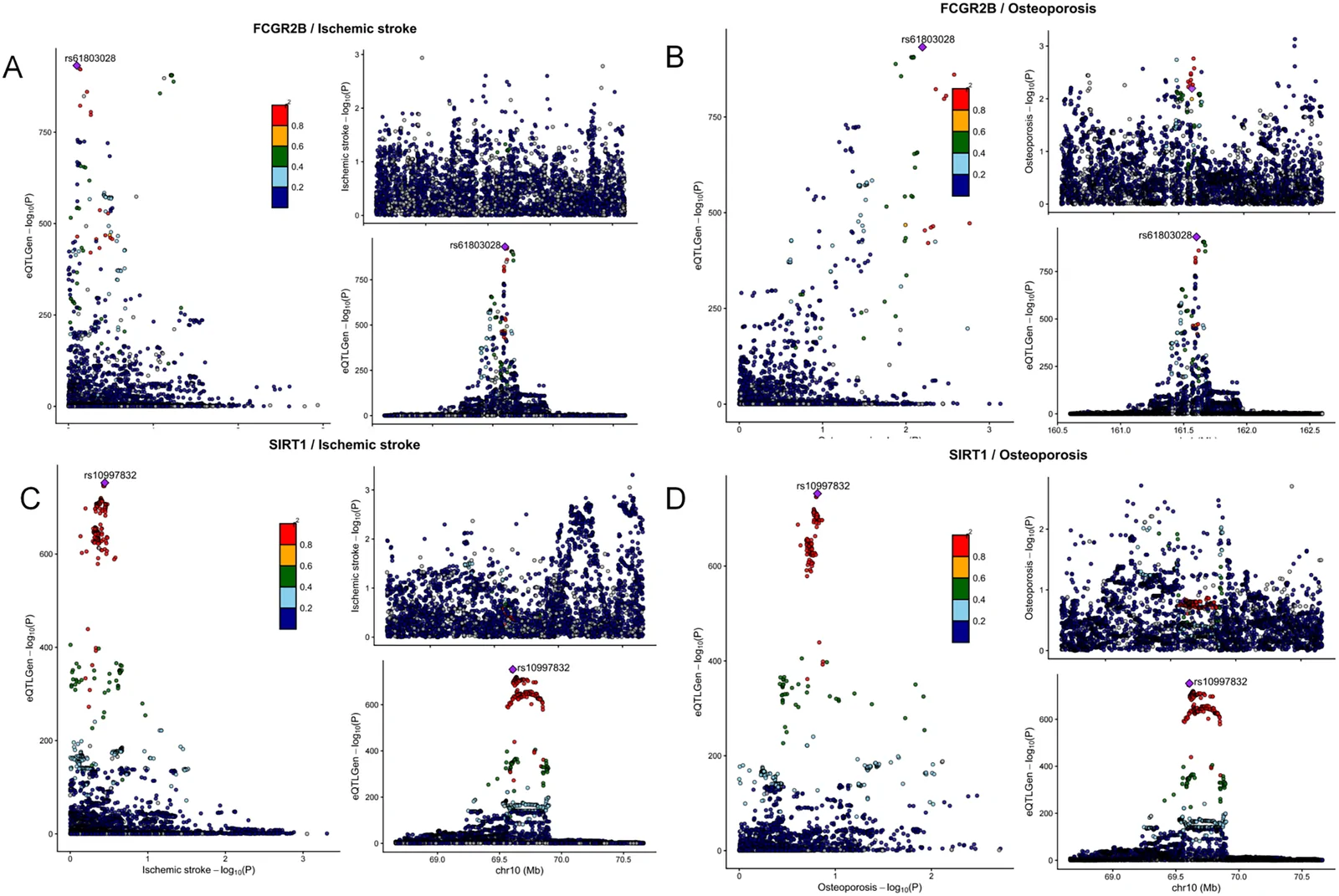
Colocalization analysis of candidate genes for ischemic stroke and osteoporosis **(A, B)** Regional colocalization plot of the *FCGR2B* locus based on GWAS summary statistics for ischemic stroke and osteoporosis. The peak variant rs61803028 yields near-complete SNP.PP.H4 for both phenotypes. **(C, D)** Regional colocalization plot of the *SIRT1* locus. The top variant rs10997832 shows moderate SNP.PP.H4 of 0.5503 for ischemic stroke and 0.5489 for osteoporosis, suggesting dispersed posterior probability distribution across this locus. SNP.PP.H4 refers to the posterior probability that the corresponding SNP is the shared causal variant under hypothesis H4.

Together with uncorrected nominal MR screening results, these colocalization outputs further support these genes as exploratory candidates rather than definitively proven causal mediators.

### Signaling pathways involved in key genes

GSVA revealed that, in ischemic stroke data, *CYBB* was enriched in pathways such as REACTIVE_OXYGEN_SPECIES_PATHWAY, P53_PATHWAY, and PI3K_AKT_MTOR_SIGNALING (**Figure 5A**). *FCGR2B* was enriched in TGF_BETA_SIGNALING and TNFA_SIGNALING_VIA_NFKB (**Figure 5B**), while *SIRT1* was associated with MTORC1_SIGNALING and IL2_STAT5_SIGNALING (**Figure 5C**).

**Figure 5:**
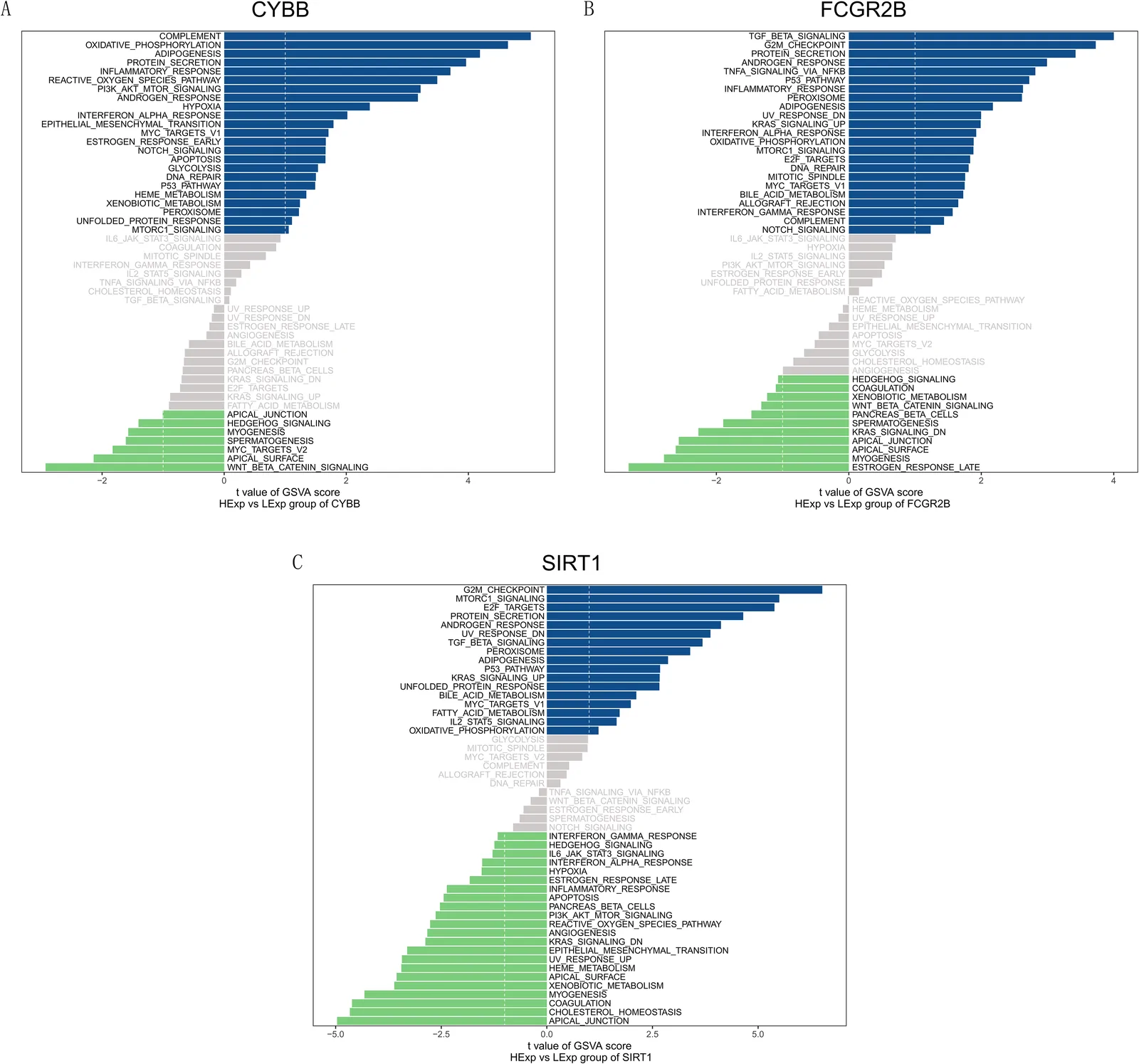
Signaling pathways involved in key genes of IS. **(A-C)** GSVA enrichment bar charts *CYBB, FCGR2B*, and *SIRT1*, respectively. Blue, grey and green bars correspond to biological process, cellular component, and molecular function ontology terms. X-axis indicates enrichment score, and labels list enriched functional terms, illustrating potential biological mechanisms underlying ischemic stroke-osteoporosis comorbidity.

In osteoporosis, *CYBB* was enriched in pathways including ANDROGEN_RESPONSE and HEME_METABOLISM (**Figure 6A**), *FCGR2B* in PROTEIN_SECRETION and MYC_TARGETS_V1 (**Figure 6B**), and *SIRT1* in ANDROGEN_RESPONSE and E2F_TARGETS (**Figure 6C**).

**Figure 6:**
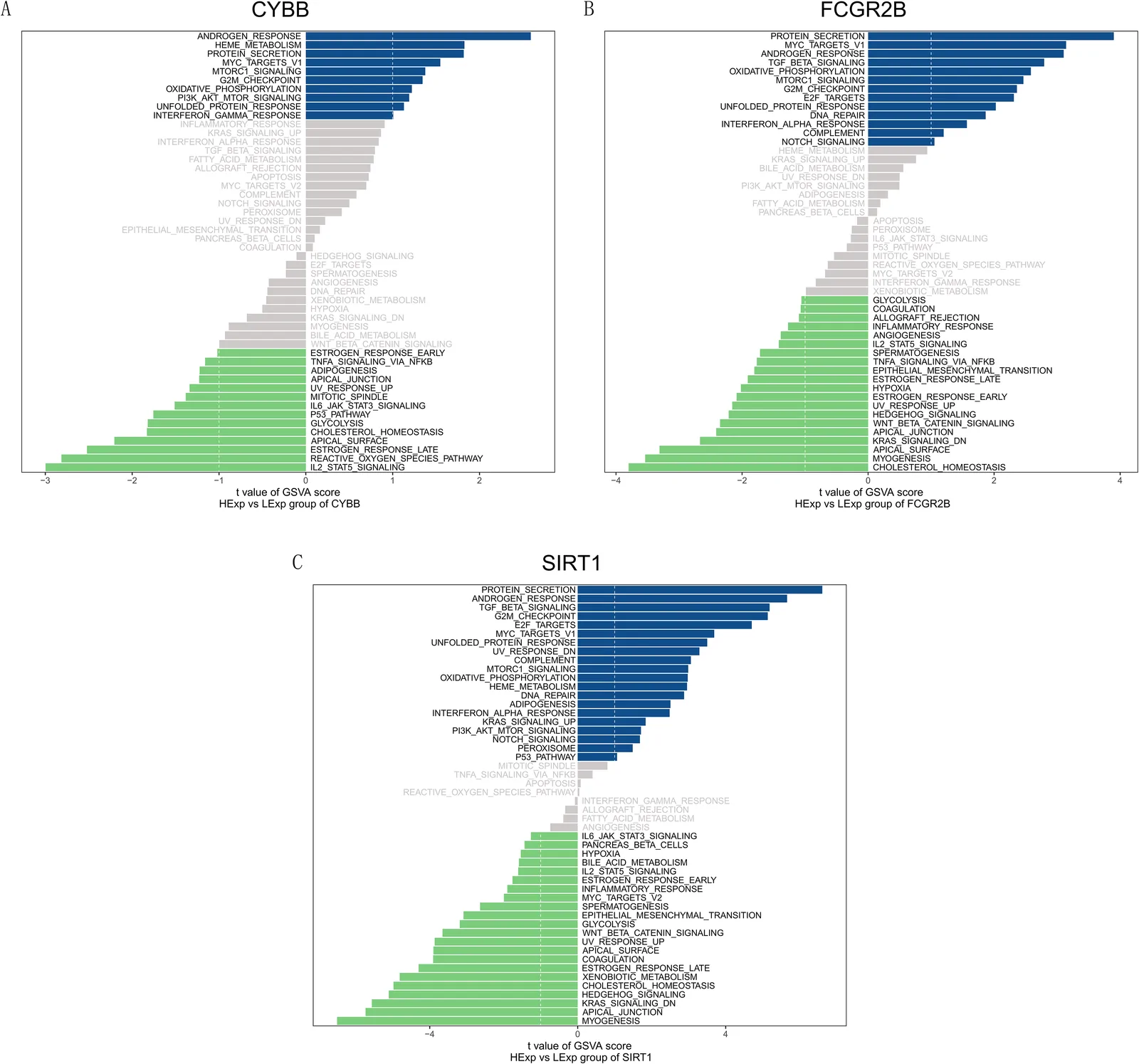
Signaling pathways involved in key genes of osteoporosis. **(A)** *CYBB*, **(B)** *FCGR2B*, **(C)** *SIRT1*. X-axis shows t-values of GSVA scores between HExp and LExp groups. Blue: high-expression-activated pathways; green: low-expression-enriched pathways; gray: non-significant pathways. The results illustrate differential signaling regulation patterns of the three genes during ischemic stroke-osteoporosis comorbidity.

Complementary Gene Set Enrichment Analysis (GSEA) revealed additional signaling mechanisms. In ischemic stroke, *CYBB* was enriched in NOD-like receptor and PPAR signaling pathways (**Figure 7A**), *FCGR2B* in p53 and Toll-like receptor signaling (**Figure 7B**), and *SIRT1* in RNA degradation and Selenocompound metabolism (**Figure 7C**). In osteoporosis, enrichment included Necroptosis and Nucleocytoplasmic transport for *CYBB* (**Figure 8A**), *FCGR2B* (**Figure 8B**) and basal transcription and spliceosome-related pathways for *SIRT1* (**Figure 8C**).

**Figure 7:**
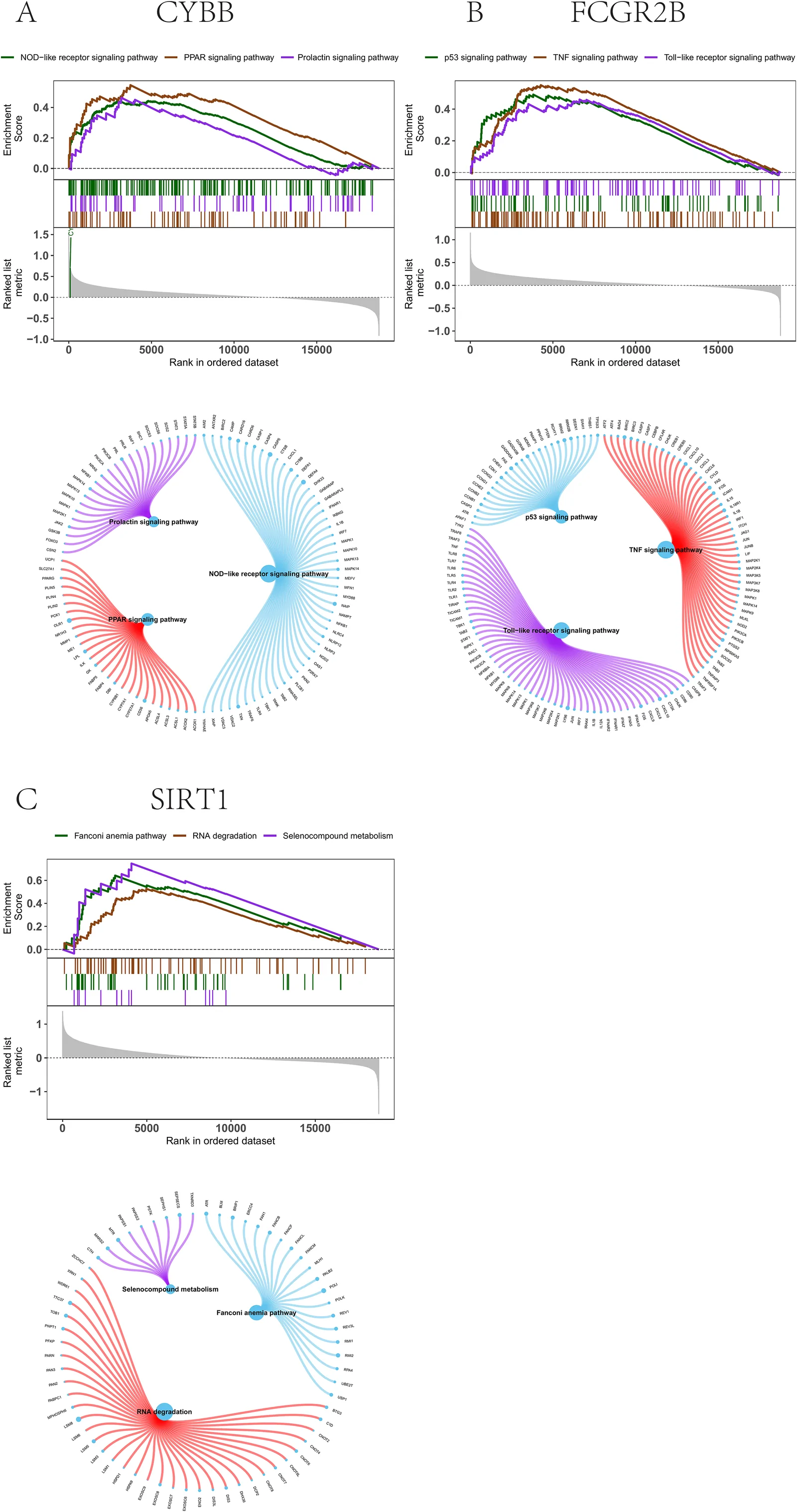
Gene set enrichment analysis (GSEA) of three key genes in ischemic stroke. **(A)** *CYBB*, **(B)** *FCGR2B*, **(C)** *SIRT1*. Each panel contains a GSEA enrichment curve plot and a supporting chord diagram. Enrichment score and ranked gene distribution are presented in line charts; chord diagrams quantify gene-pathway associations. Multiple immune-related and metabolic signaling pathways are significantly enriched for the three genes, clarifying their potential functional roles in ischemic stroke.

**Figure 8:**
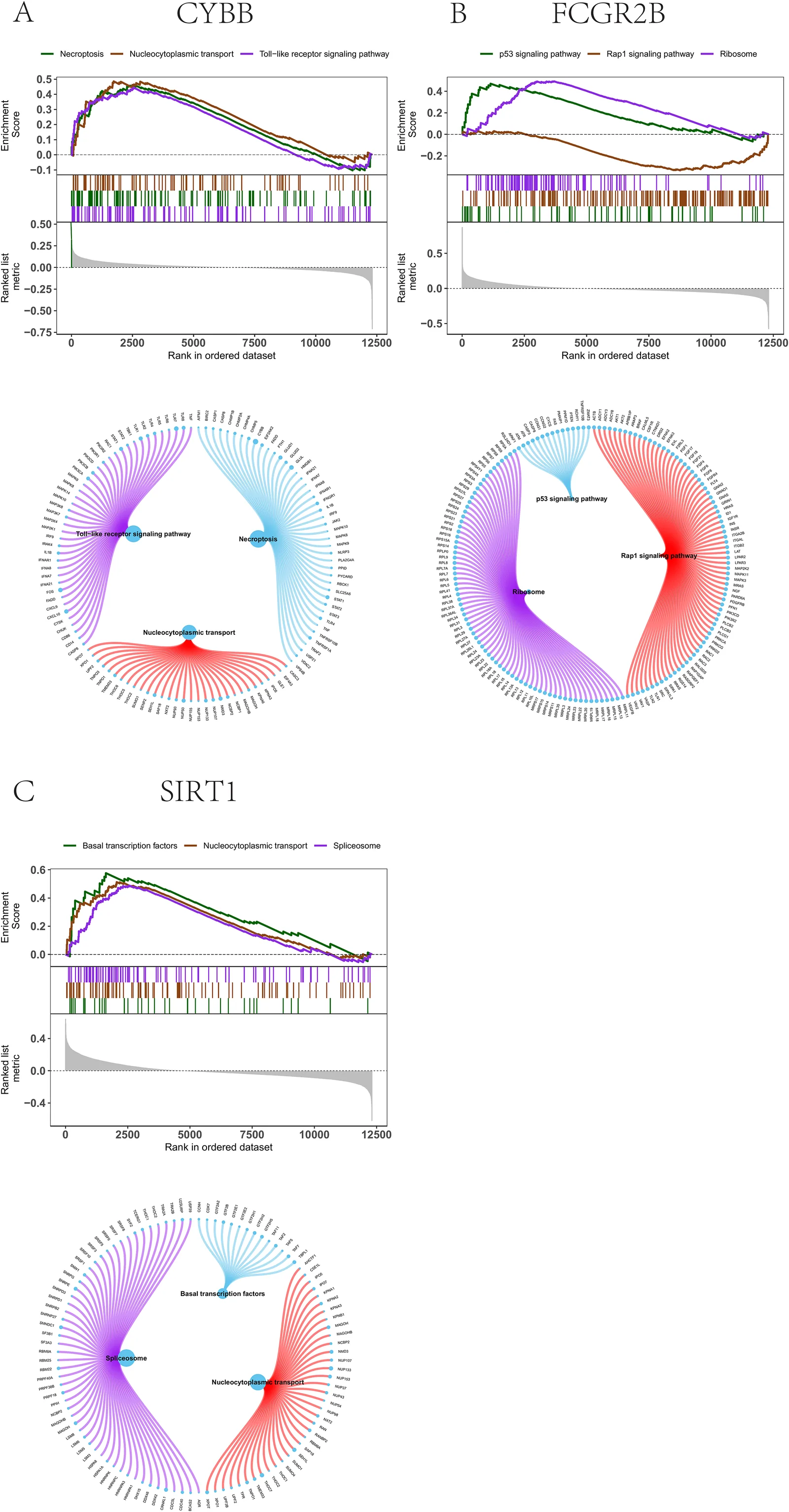
GSEA of OP. **(A)** *CYBB*, **(B)** *FCGR2B*, **(C)** *SIRT1*. Each panel consists of a GSEA enrichment curve plot and a matching chord diagram. Line charts display enrichment score dynamics and ranked gene distribution; chord diagrams quantify gene-pathway correlations. Multiple functional signaling pathways are significantly enriched for the three candidate genes, revealing their potential regulatory mechanisms in osteoporosis pathogenesis.

### Immune infiltration analysis

Immune infiltration analysis showed significant differences in immune cell populations between cases and controls for both diseases (**Figures 9A–C**, **10A-C**). In ischemic stroke, immune components such as APC_co_stimulation, macrophages, neutrophils, and Tregs were elevated in cases (**Figure 9C**). Correlation analyses revealed strong relationships between key genes and immune cells. For instance, *SIRT1* was positively associated with Th2 cells and aDCs but negatively correlated with APC_co_inhibition and CCR. *FCGR2B* showed positive correlations with APC_co_stimulation and Tregs, while *CYBB* was strongly positively correlated with macrophage and neutrophil enrichment scores (exact correlation coefficients and adjusted *P*-values for all major immune cell signatures are provided in **Supplementary Tables S2**, **3**) (**Figure 9D**). Consistently, our single-cell RNA-seq profiling independently verified that *CYBB* transcripts are preferentially enriched in monocyte and macrophage clusters at single-cell resolution. Notably, bulk immune-infiltration quantification and single-cell profiling represent two orthogonal, parallel analytical approaches; we did not perform direct statistical correlation between bulk-derived metrics and single-cell data. The matching biological patterns are interpreted as mutually supportive observational evidence from tissue-population-level and single-cell-level perspectives, respectively. Consistently, our single-cell RNA-seq profiling independently verified that *CYBB* transcripts are preferentially enriched in monocyte and macrophage clusters at single-cell resolution. The consistent positive correlation between *CYBB* expression and bulk tissue macrophage abundance matches the cell-type-specific expression pattern observed in single-cell datasets, forming mutually supportive parallel evidence from tissue population scale and single-cell resolution respectively.

**Figure 9:**
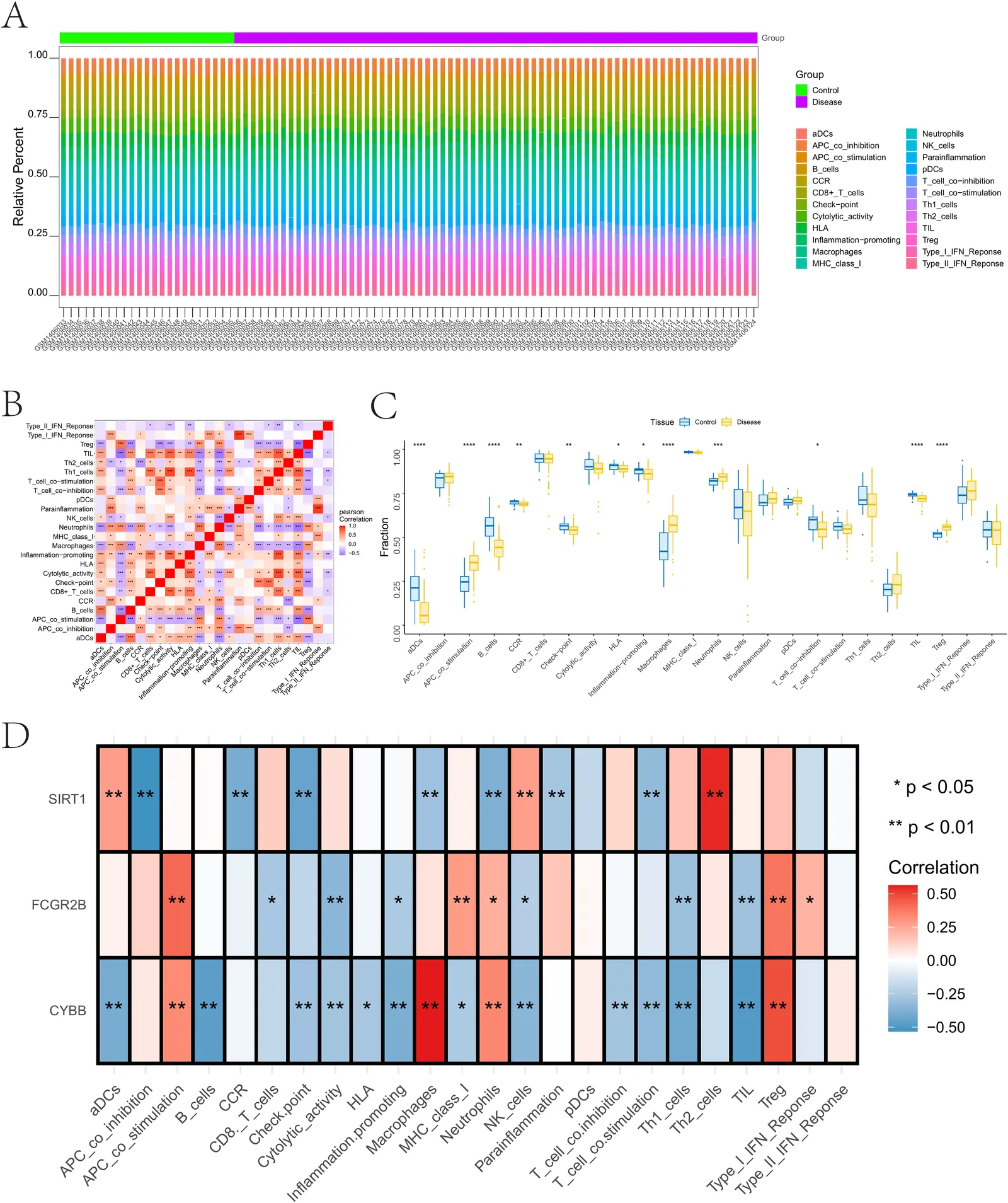
Immune infiltration analysis of IS. **(A)** ssGSEA-calculated relative abundance of immune cells across samples. **(B)** Pairwise correlation heatmap of immune-cell fractions. **(C)** Box plots comparing immune-cell infiltration between control and disease groups. **(D)** Correlation heatmap of *SIRT1, FCGR2B, CYBB* with infiltrating immune cells. *p* < 0.05, *p* < 0.01. The figure depicts immune microenvironment features and gene-immune crosstalk in ischemic stroke.

**Figure 10:**
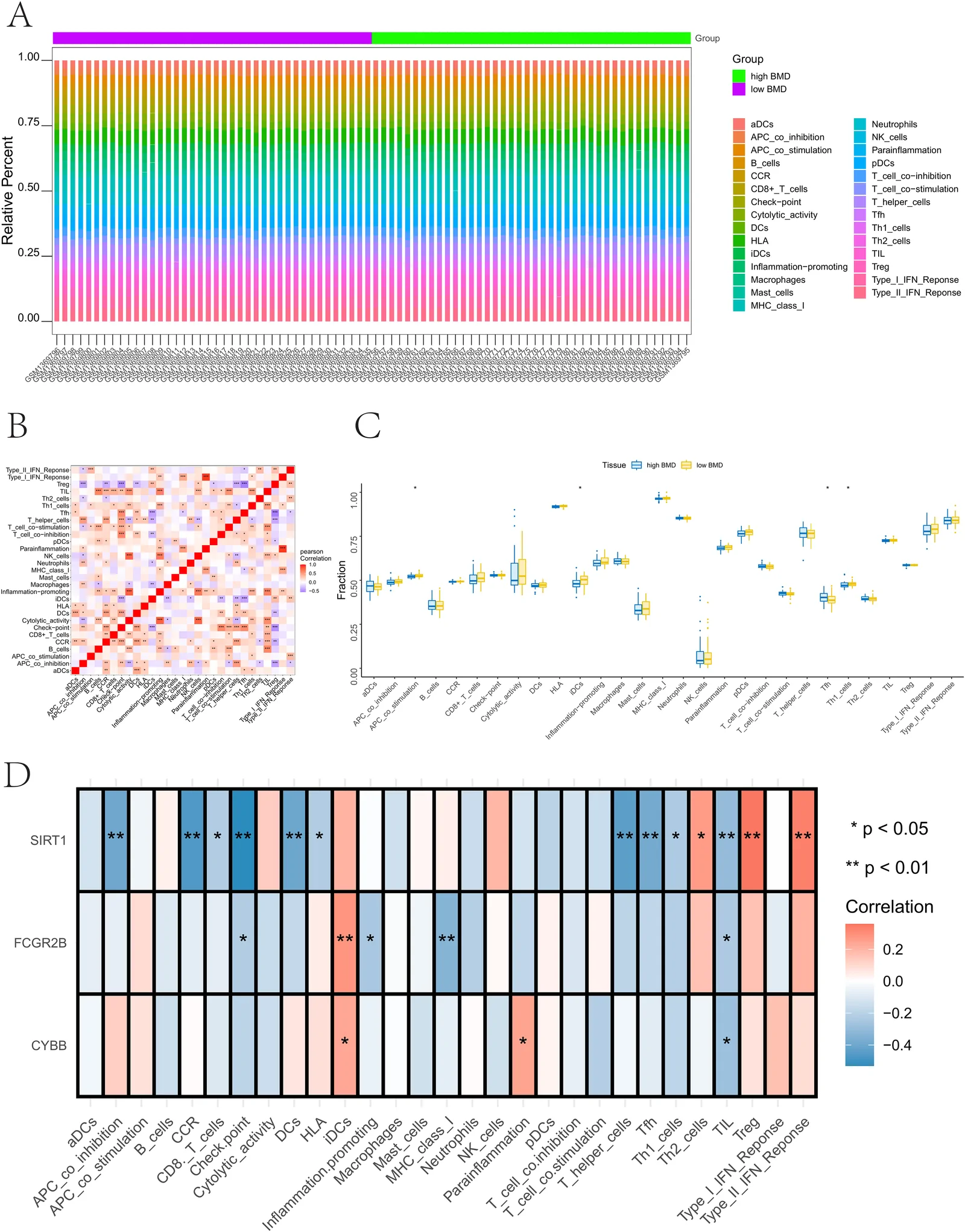
Immune infiltration analysis of OP (high-BMD vs low-BMD subgroups of the GSE56815 cohort). **(A)** ssGSEA-derived relative abundance of immune cells across high-/low-BMD samples. **(B)** Pairwise correlation heatmap of infiltrating immune cell fractions. **(C)** Box plots for intergroup comparison of immune cell infiltration levels. **(D)** Correlation between *SIRT1, FCGR2B, CYBB* and immune cell subsets (*p* < 0.05, *p* < 0.01). This figure illustrates immune microenvironment features and hub-gene-immune interactions in osteoporosis populations with different bone mineral density.

In osteoporosis, significant differences were observed for APC_co_stimulation, iDCs, Tfh, and Th1 cells (**Figure 10C**). *SIRT1* was positively correlated with Type_II_IFN_Reponse and Tregs but negatively associated with T helper cells and immune checkpoints (**Figure 10D**). *FCGR2B* was positively associated with iDCs and negatively with inflammation-promoting markers, while *CYBB* showed similar patterns. These findings highlight the involvement of key genes in modulating the immune microenvironment in both diseases.

### Transcriptional regulation analysis

Motif-based TF enrichment analysis identified shared regulatory mechanisms for the key genes. The motif with the highest normalized enrichment score (NES = 4.12) was cisbp:M1399. All enriched motifs and their corresponding TFs are summarized in **Figure 11**, indicating potential upstream regulatory factors involved in disease pathogenesis.

**Figure 11:**
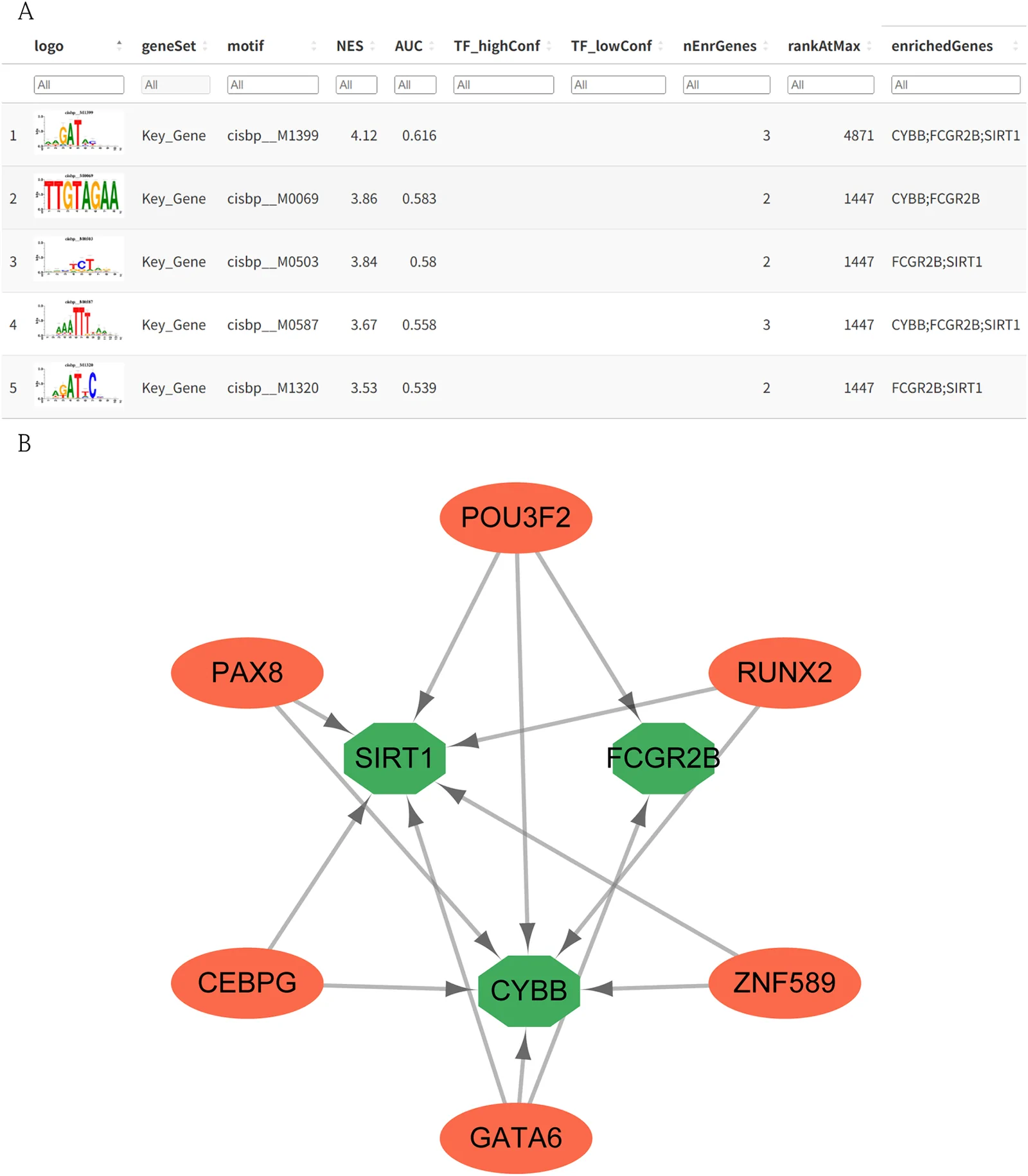
Motif-based TF enrichment analysis. **(A)** Enrichment statistics of candidate motifs paired with quantitative indexes such as NES and AUC. **(B)** TF-hub gene regulatory network: upstream transcription factors (red nodes) show directional regulatory effects on *SIRT1, FCGR2B* and *CYBB* (green nodes). The network interprets the upstream transcriptional regulation patterns of the three core genes.

### Analysis of disease regulation of key genes

1. Ischemic stroke: This study obtained the disease-regulated genes of ischemic stroke through the GeneCards database (https://www.genecards.org/). This study analyzed the expression levels of the top 20 genes in the Relevance score and found that F5, NOS3, ALOX5AP, and COL4A1 were significantly different between the two groups. In addition, the expression levels of the three key genes and the expression levels of disease regulatory genes were significantly different. There is a significant correlation, among which *CYBB* and F5 are significantly positively correlated (cor=0.524, p=8.1e−08), and *CYBB* and PRKCH are significantly negatively correlated (cor=-0.4, p=7.9e−05) (**Figure 12A**).
2. Osteoporosis: This study obtained the disease-regulating genes of osteoporosis through the GeneCards database (https://www.genecards.org/). This study analyzed the expression levels of the top 20 genes in Relevance score and found that LRP5, COL1A1, WNT1, VDR, CALCR, DKK1, ESR1, BGLAP, TNFSF11, and RUNX2 were significantly different between the two groups. In addition, 3 key genes The expression levels of genes are significantly correlated with the expression levels of disease regulatory genes, among which *SIRT1* and *COPB2* are significantly positively correlated (cor=0.505, p=1.8e−06), and *SIRT1* and *ESR1* are significantly negatively correlated (cor=-0.523, p =6.4e− 07) (**Figure 12B**).

**Figure 12:**
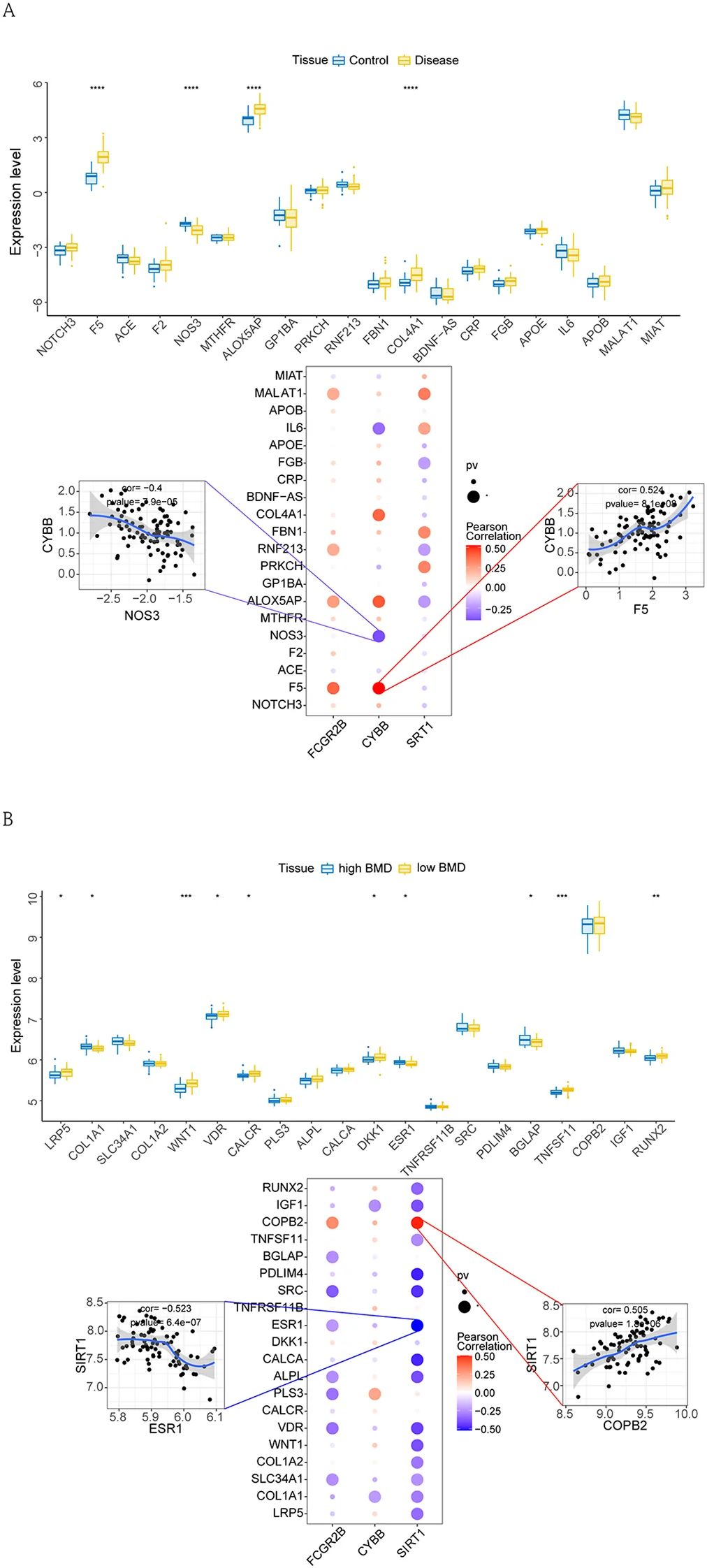
the disease-regulated genes. **(A)** Gene differential expression and gene-gene correlation in ischemic stroke cohorts; representative scatter plots show correlation trends of *CYBB* with target genes. **(B)** Differential expression and correlation patterns of disease-regulated genes in high-/low-BMD osteoporosis subgroups, with exemplary linear correlations of *SIRT1* with *ESR1* and *COPB2*. Red/blue in bubble heatmaps indicate positive/negative Pearson correlations, revealing downstream regulatory partners of the three hub genes in IS-OP comorbidity. *P < 0.05, **P < 0.01, ***P < 0.001, ****P < 0.0001.

### Single cell analysis

1) Ischemic stroke: This analysis uses GSE174574 single cell data, including 6 samples, including 3 control samples and 3 disease samples. Only nFeature_RNA > 1000 & percent.mt < 5 & in the expression profile are retained. Cells with nFeature_RNA < 6000 & nCount_RNA < 30000 were used for this analysis, and the feature expression levels of a total of 45,163 cells were included for subsequent analysis. Data were standardized and homogenized to reduce technical bias. This study applied the t-distributed stochastic neighbor embedding (tSNE) method to further dimensionality reduction and visual analysis of the data, and 15 subtypes were obtained through tSNE at a resolution of 0.2 (**Figure 13A**). Each subtype was annotated through the R package SingleR, and 15 clusters were annotated into 10 cell categories: Endothelial cells, Microglia, Monocytes, Astrocytes, Epithelial cells, Macrophages, Oligodendrocytes, Granulocytes, Fibroblasts, and NK cells (**Figure 13B**). This study also explored the expression of *Fcgr2b, Cybb* and *Sirt1* in 10 cell categories: Endothelial cells, Microglia, Monocytes, Astrocytes, Epithelial cells, Macrophages, Oligodendrocytes, Granulocytes, Fibroblasts, and NK cells (**Figures 13C–E**). Of note, the mouse-derived ischemic-stroke single-cell dataset GSE174574 contains only three animals per group, and observations from mouse brain tissue should be interpreted cautiously when extrapolated to human ischemic-stroke pathophysiology.

**Figure 13:**
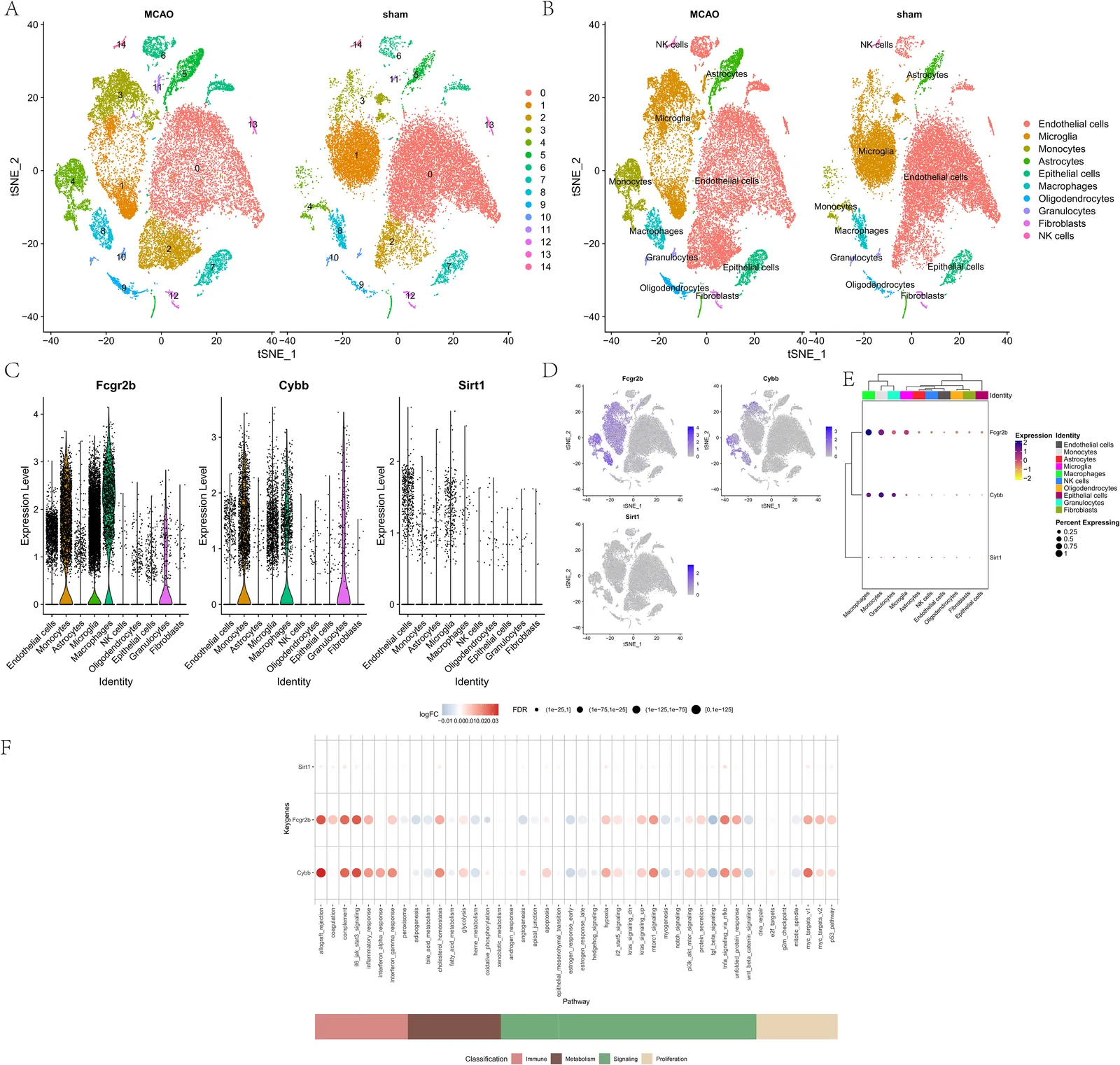
Single cell analysis of IS. **(A, B)** t-SNE plots of annotated cell clusters from MCAO and sham groups. **(C)** Violin plots showing *Fcgr2b, Cybb, Sirt1* expression across cell types. **(D)** Feature UMAPs for hub-gene expression. **(E)** Dot plot of cell-cluster marker genes. **(F)** Bubble plot of pathway activity scores in MCAO versus sham. Single-cell data delineate cell-type-specific expression signatures of the three hub genes in ischemic stroke.

In addition, this study used the AUCell function to calculate the enrichment scores of different immune/metabolic pathways for each cell.Then, the cells were divided into two groups based on the median expression of *FCGR2B*, *CYBB*, and *SIRT1*. We detected differences in enrichment scores of immune/metabolic pathways within the osteoporosis (OP) single-cell dataset. Given that this dataset was derived from only one OP patient, these findings should be interpreted cautiously with limited generalizability and represent only exploratory single-case observations. Differences in enrichment scores of immune/metabolic pathways (**Figure 13F**). It also shows the gene co-expression of the three genes with the highest Relevance score among the disease-regulated genes and key genes, as well as the correlation of co-expressed genes (**Supplementary Materials 4**–**6**).

2) Osteoporosis: This analysis utilized the human bone marrow mesenchymal stem cell scRNA-seq dataset GSE147287 deposited in the GEO database, which only contains one patient with osteoporosis and one patient with osteoarthritis as biological samples. Given the extremely limited sample size restricted to a single OP case, we pooled all cells isolated from this sole osteoporosis patient for subsequent dimensional reduction and cell-type annotation to maximize the number of high-quality cells (8,492 cells retained) for exploratory cell-type expression profiling. We acknowledge that scRNA-seq data derived from only one osteoporosis individual cannot capture population-level inter-individual heterogeneity, and all cell-type expression patterns observed herein should be interpreted strictly as exploratory single-case observational results rather than generalizable population-level conclusions.The feature expression levels were included for subsequent analysis. Data were standardized and homogenized to reduce technical bias. This study applied the t-distributed stochastic neighbor embedding (tSNE) method to further dimensionally reduce and visualize the data, and obtained 15 subtypes through tSNE at a resolution of 0.2 (**Figure 14A**). This study used the R package SingleR to annotate each subtype, and 15 clusters were annotated to 11 cells: Tissue_stem_cells, Neutrophils, Myelocyte, Macrophage, Monocyte, T_cells, BM, Erythroblast, Pre−B_cell_CD34−, B_cell, and Pro−B_cell_CD34+(**Figure 14B**).We further performed exploratory profiling of *FCGR2B, CYBB* and *SIRT1* expression across the 11 annotated cell subpopulations derived from this single osteoporosis patient’s bone marrow mesenchymal cells, with results limited to case-specific observational patterns: Tissue_stem_cells, Neutrophils, Myelocyte, Macrophage, Monocyte, T_cells, BM, Erythroblast, Pre−B_cell_CD34−, B_cell, and Pro−B_cell_CD34+ (**Figures 14C–E**). In addition, this study used the AUCell function to calculate the enrichment scores of different immune/metabolic pathways for each cell. It also shows the gene co-expression of the three genes with the highest Relevance score among the disease-regulated genes and key genes, as well as the correlation of co-expressed genes (**Supplementary Materials 7**-**10**).

**Figure 14:**
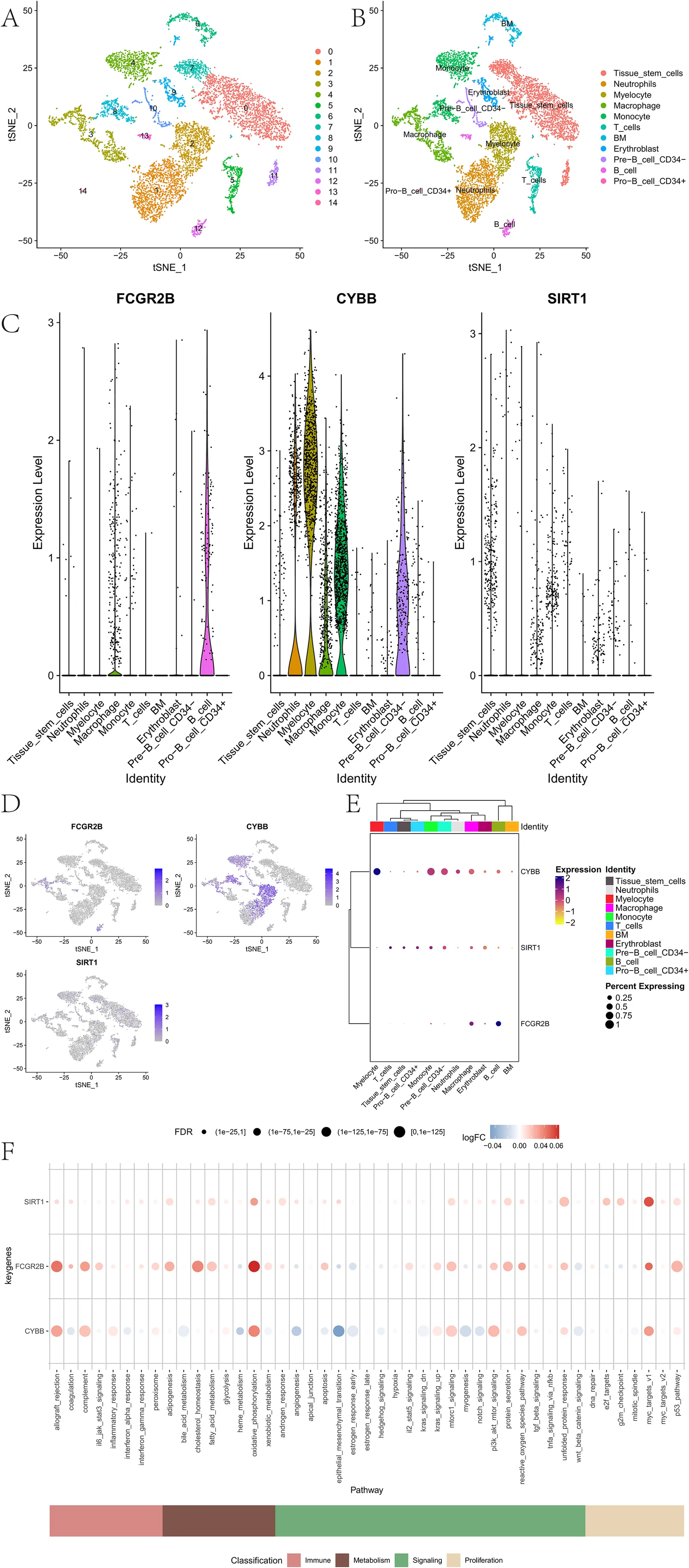
Single cell analysis of OP. **(A, B)** UMAP visualization of annotated cell clusters from osteoporosis patient tissue. **(C)** Violin plots of *FCGR2B, CYBB, SIRT1* expression across cell subsets. **(D)** UMAP-based feature plots for hub-gene expression. **(E)** Dot plot of cell-cluster marker genes.

This cell-type-specific enrichment pattern of *CYBB* in macrophages is consistent with our bulk transcriptome immune infiltration analysis, which identified a robust positive correlation between overall *CYBB* expression level and macrophage infiltration abundance across patient tissue samples. The two analytical approaches provide complementary parallel evidence from distinct biological scales.

### Experimental validation of co-susceptible genes

We successfully constructed a mouse model of IS/OP comorbidity, and validated it through Micro-CT examination and TTC **(Supplementary Materials 1**-**3**). The expression levels of the shared molecules were validated in a mouse model. Analysis of gene expression in mouse blood revealed that the mRNA levels of Fcgr2b (*P* < 0.001) and Cybb(*P* < 0.0001) in the model group were markedly increased compared to the control group, whereas the mRNA levels of Sirt1 were significantly decreased (*P* < 0.05) (**Figure 15**). Of note, qPCR analyses were performed using archived RNA aliquots with an effective biological sample size of n = 4 per group due to sample damage; reported qPCR replicates are technical replicates on stored RNA, rather than independent biological replicates from newly-isolated animal material. It should be clarified that this transcriptional alteration in circulating blood only serves as auxiliary supporting evidence for the shared gene signature screened from human multi-omics datasets, rather than definitive proof distinguishing gene regulatory effects induced by isolated cerebral ischemia, isolated osteoporosis, or their synergistic comorbid interaction. The core evidence identifying *FCGR2B*, *CYBB* and *SIRT1* as shared pathogenic genes relies on human two-sample MR-GWAS, blood eQTL integration and cell-type-specific single-cell transcriptomic profiling of brain and bone tissues. The absence of IS-only and OP-only mouse groups restricts us from fully disentangling disease-specific transcriptional drivers at the animal level, and these mouse blood data alone cannot independently verify the shared-gene conclusion.

**Figure 15:**
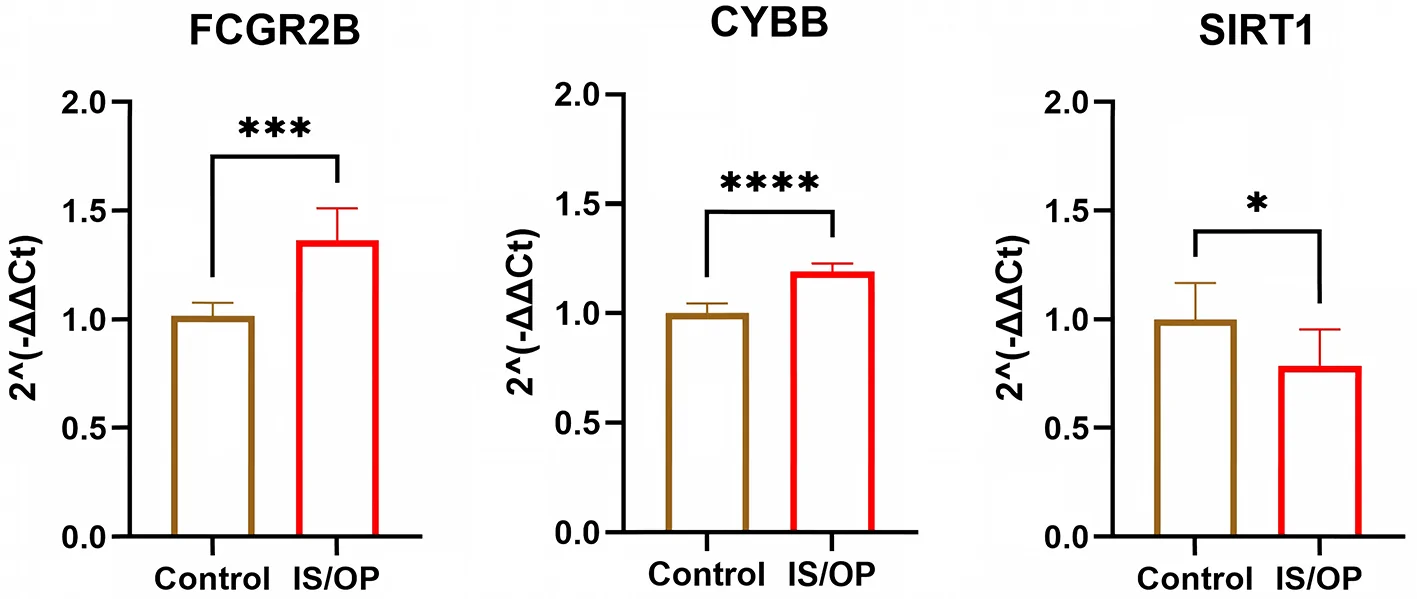
Analysis of gene expression in mouse blood revealed that the mRNA levels of Fcgr2b (*P* < 0.01) and Cybb(*P* < 0.001) in the model group were markedly increased compared to the control group, whereas the mRNA levels of Sirt1 were significantly decreased(technical replicates on archived RNA). qPCR was performed on archived RNA aliquots; effective biological sample size n = 4 per group due to sample damage, and repeated qPCR measurements represent technical replicates instead of independent biological animal replicates. Relative expression was calculated via 2^ΔΔCt method using the mean ΔCt of all control samples as calibrator (control group average = 1). Individual replicates naturally fluctuate around the normalized mean. Raw Cq values are provided in **Supplementary Table S1**, amplification-melting curves are shown in **Supplementary Figure S11**. *P < 0.05, **P < 0.01, ***P < 0.001, ****P < 0.0001.

### Functional validation of SIRT1 in post-stroke osteoporosis mice

To explore the function of *SIRT1* in post-stroke osteoporosis, the expression of *SIRT1* was intervened by AAV vectors. Western blot analysis confirmed that *SIRT1* protein levels were significantly reduced in *SIRT1* knockdown group compared to the control group, whereas *SIRT1* overexpression vector infection markedly increased its expression (**Supplementary Material 11**). Intracerebroventricular injection of AAV-*SIRT1* (OE) successfully restored *SIRT1* protein expression in the prefrontal cortex, which subsequently mitigated ischemic brain injury and reduced focal neuroinflammation. Intriguingly, this central neuroprotection was accompanied by a significant systemic improvement in bone turnover, as evidenced by elevated serum ALP levels. In mice serum, *SIRT1* knockdown significantly reduced ALP levels and increased CTX-1 levels compared to the PBS control group, as shown in **Table 2**. Conversely, *SIRT1* overexpression resulted in elevated ALP levels and decreased CTX-1 levels compared to the PBS control group. These serum marker results provide preliminary associative hints that central *SIRT1* modulation may correlate with altered peripheral bone turnover-related serum markers under conditions of stroke-osteoporosis comorbidity. Direct mechanistic evidence linking central *SIRT1* activity to peripheral bone metabolism remains absent, given the lack of IS-only and OP-only control groups. These observational biomarker changes should be interpreted cautiously, and definitive causal inference relies primarily on human multi-omics evidence rather than the present animal data. However, without IS-only and OP-only animal groups, we cannot confirm whether this regulatory effect is specifically induced by stroke, osteoporosis, or their combination; definitive mechanistic inference still relies on human multi-omics evidence rather than animal peripheral biochemical readouts.

**Table 2:** Changes in ALP and CTX-1 levels in mice (n=5).

Group	ALP(U/L)	CTX-1(ng/mL)
PBS	89.64 ± 3.02	47.52 ± 0.96
KD	35.73 ± 1.95**	50.67 ± 2.35*
OE	94.55 ± 1.34*	24.98 ± 3.48*

Compared with the PBS group, *P < 0.05, **P < 0.01.

## Discussion

By integrating multi-omics data, this study highlights three exploratory MR-supported candidate genes (*FCGR2B, CYBB, SIRT1*) with nominal genetic suggestive associations with both ischemic stroke (IS) and osteoporosis (OP) (17–19). All three genes converge on overlapping immune-metabolic signaling pathways that participate in the pathological processes of the two aging-related diseases (20–23). This finding helps explain the frequent co-occurrence of IS and OP in elderly populations and identifies potential targets for developing dual intervention strategies.

### Correlation analyses revealed strong relationships between key genes and immune cell populations

For instance, *SIRT1* was positively associated with Th2 cells and aDCs but negatively correlated with APC_co_inhibition and CCR; *FCGR2B* exhibited positive correlations with APC_co_stimulation and Tregs, while *CYBB* showed a robust positive correlation with macrophage and neutrophil infiltration scores (Pearson r > 0.4, *P* < 0.01). Notably, single-cell RNA-seq and bulk transcriptome-based immune infiltration deconvolution capture immune features at distinct biological scales, which endows the two analytical pipelines with complementary, parallel interpretative value (24). Single-cell profiling delineates cell-type-intrinsic gene expression patterns at cellular resolution, directly pinpointing which immune subsets inherently transcribe target genes, whereas ssGSEA and CIBERSORTx quantify the overall proportion of immune populations within heterogeneous whole tissue samples, reflecting tissue-level immune landscape shifts under disease stress (25). Consistent with this cross-scale matching, our single-cell results independently verified prominent enrichment of *Cybb* transcripts in monocyte and macrophage clusters, which aligns perfectly with the bulk-level positive association between global *CYBB* expression and macrophage abundance across patient cohorts. The parallel concordance between single-cell cell-type enrichment and bulk immune infiltration correlation achieves reciprocal cross-validation, jointly supporting the dominant role of macrophage-expressed *CYBB* in the pathological progression of both ischemic stroke and osteoporosis (26).

It should be emphasized that MR analysis infers potential causality between genetically predicted gene expression and disease risk, but does not directly identify shared disease GWAS loci. The eQTL SNPs utilized as MR instruments in this study are not necessarily overlapping risk variants for IS and OP. Therefore, *FCGR2B, CYBB* and *SIRT1* must be interpreted strictly as exploratory MR-supported putative candidate genes, and further multi-omics and experimental validations are still required to verify their biological roles in IS-OP comorbidity (27, 28).

### Integration with traditional pathophysiological concepts

The rapid bone loss post-stroke, often attributed primarily to mechanical disuse, may be synergistically and profoundly accelerated by the concurrent molecular storm: downregulation of protective *SIRT1* signaling (impairing vascular and anabolic capacity) coupled with *CYBB/FCGR2B*-driven inflammation and oxidative stress (enhancing catabolic signaling). This framework integrates neuroendocrine stress, systemic inflammation, and local bone metabolism into a coherent systemic narrative (29).

Therefore, our findings should be regarded as suggestive rather than conclusive. Future mechanistic studies are crucial to elucidate how these genes interact under co-occurring conditions—for example, investigating whether *SIRT1*’s metabolic effects in bones indirectly influence cerebrovascular health through systemic inflammation. Longitudinal clinical cohort studies could further explore how regulating these targets affects the shared pathogenesis of both diseases (30, 31).

Single-cell transcriptomics and bulk immune deconvolution assays capture immune features at different biological dimensions: single-cell data pinpoints intrinsic gene expression within individual cell subsets, whereas bulk immune infiltration metrics quantify total cell population proportions in heterogeneous tissues. In this study, the macrophage-specific enrichment of *CYBB* observed in single-cell analysis was cross-validated via positive correlation between *CYBB* transcriptional levels and macrophage infiltration scores in bulk patient datasets. These two lines of evidence corroborate each other while remaining parallel, independently supporting the conclusion that *CYBB* exerts its pathological function mainly through tissue macrophages in both ischemic stroke and osteoporosis lesions (32, 33).

In conclusion, by integrating genetic evidence with cellular and pathway-level analyses, we propose a preliminary framework: IS and OP share overlapping immune-metabolic pathological mechanisms. The three exploratory MR-supported candidate genes *FCGR2B*, *CYBB* and *SIRT1* may plausibly participate in the pathological progression of both diseases, yet definitive causal roles remain to be established.

### Limitation

This study has several important limitations that should be acknowledged when interpreting its findings.

First, the human multi-omics analyses in this work are observational in nature. GWAS and Mendelian randomization outputs provide evidence for genetic association and putative causal inference, yet they cannot directly confirm biological causality between candidate genes and IS-OP comorbidity.Although we implemented Bayesian colocalization to interrogate shared causal variants, moderate posterior probability for *SIRT1* suggests inconclusive evidence for shared causal variants at this locus. We still cannot fully rule out linkage disequilibrium as a potential contributor to the observed MR association signals. Direct experimental evidence including genome-wide co-expression network profiling, protein-protein interaction assays, and multi-gene intervention animal models is still absent to verify potential coordinated functional crosstalk among *FCGR2B, CYBB*, and *SIRT1*. Although these genes share upstream transcription factors and converge on overlapping immune-metabolic pathways for IS and OP, their physical interactions and synergistic crosstalk remain unvalidated. In particular, the gene-level MR screening relied on nominal uncorrected *P*-values, and no gene achieved significance following Benjamini-Hochberg FDR adjustment across the 4463 druggable genes; our candidate genes therefore remain exploratory until further mechanistic replication (34).

Second, substantial cross-species and translational gaps exist between human-derived datasets and mouse-based *in-vivo* experiments. Our combined aging-MCAO mouse model develops bone loss via aging-dependent bone deterioration plus post-stroke disuse, whereas human IS-related osteoporosis is modulated by multiple complex confounders such as medication, nutritional status, and heterogeneous clinical disease courses. Thus, mouse experimental observations cannot be fully extrapolated to human IS-OP comorbidity. Furthermore, our animal validation only includes comorbid IS/OP and healthy control groups, lacking independent IS-only and OP-only arms. In addition, we lack direct mechanistic evidence illustrating how central SIRT1 intervention modulates peripheral bone turnover markers; serum biomarker changes in our animal experiment only reflect preliminary associative observations rather than proven causal mechanistic links. And formal quantitative analyses including cerebral HE/TUNEL staining for ischemic injury, bone trabecular histological staining and TRAP staining for osteoclast counting are not included in the current work due to experimental constraints. This prevents us from distinguishing whether gene expression changes stem from cerebral ischemia, osteoporosis, or their combined pathological interaction; accordingly, peripheral-blood qPCR results from mice serve merely as secondary circulatory-level reference rather than definitive mechanistic proof, and robust inference of shared disease genes mainly relies on multi-layer human genetic and transcriptomic evidence.

Third, sample-size constraints are present across multiple datasets. For the ischemic-stroke single-cell dataset GSE174574, only 3 control and 3 MCAO-disease mouse samples were included; the relatively small sample size may limit statistical power for detecting subtle cell-type expression alterations. The public osteoporosis single-cell dataset GSE147287 is human-derived bone-marrow mesenchymal stem-cell data containing only one OP patient. Expression signatures of *FCGR2B*, *CYBB* and *SIRT1* identified from pooled cells of this single case cannot represent population-level transcriptomic heterogeneity among osteoporosis patients. Additional single-cell profiling with multiple independent patient samples is needed to validate these cell-level patterns (35).

Fourth, tissue mismatch constitutes a key methodological limitation. Core gene-screening analyses depend on peripheral-blood GWAS and blood cis-eQTL data that capture regulatory effects only in circulating immune cells, while pathological lesions of IS and OP occur in brain parenchyma and bone microenvironment. Owing to pronounced tissue- and cell-type-specific gene regulation, blood-detected genetic signals may not reflect transcriptional behavior within brain and bone target tissues. Although we used brain and bone-marrow single-cell data to localize candidate-gene expression in disease-relevant cell subsets, matched human brain- and bone-derived eQTL datasets are currently scarce, limiting further tissue-specific causal interpretation. All standardized analytical scripts for blood and tissue omics data are deposited in **Supplementary Materials** for reproducibility.

## Data Availability

The public datasets were downloaded and analyzed in this study, which can be found in eQTLGen consortium (https://www.eqtlgen.org), the FinnGen and EBI databases (finngen_R10_M13_OSTEOPOROSIS, ebi-a-GCST90018864), GENE EXPRESSION OMNIBUS (https://www.ncbi.nlm.nih.gov/geo/info/datasets.html). All used source codes of bioinformatics analysis are shown in the **Supplementary Materials**.
