# Supplementary material for: MR-supported candidate genes and pathway analysis between ischemic stroke and osteoporosis: an integrated multi-omics study
Source: Front Immunol. 2026 Oct 21;17:1926168. doi: 10.3389/fimmu.2026.1926168 (PMC13730903; doi:10.3389/fimmu.2026.1926168)
Supplement: Supplementary file 2 [file Table2.docx]

**Micro-CT showed that the bone volume fraction and trabecular thickness of the knee joint of the mice were lower than the normal values.**

Compared with the control group, the bone volume fraction and trabecular thickness of the knee joints in the model group mice showed a significantly decreased trend, while the trabecular separation degree exhibited a decreasing tendency. These changes were statistically significant (P <0.05), but the differences were not significant (P> 0.05).

**Supplementary Material 2 Micro-CT Analysis of Mice Knee Joint Tissue**（‾χ±*s，n*=3）

| Group | BV/TV | Tb.SP(mm) | Tb.Th(1/mm) | TV(mm^3^) |
| --- | --- | --- | --- | --- |
| Control Group | 0.36±0.03 | 0.12±0.04 | 0.09±0.01 | 0.95±0.04 |
| Model Group | 0.23±0.02 | 0.15±0.03 | 0.05±0.02 | 1.08±0.08 |
| *P-value* | 0.009 | 0.035 | 0.003 | 0.157 |
